# Bridging the gaps in detection of structural cardiotoxicity in stem cell-derived cardiomyocytes: promise of miR-133b, miR-184 and miR-208b-3p

**DOI:** 10.3389/fphar.2025.1584734

**Published:** 2025-07-25

**Authors:** M. Cherubin, A. Delaunois, J. P. Valentin, M. Alaerts, P. J. Guns, V. Gryshkova

**Affiliations:** ^1^ UCB Biopharma SRL, Non-Clinical Safety Evaluation, Braine-L’Alleud, Belgium; ^2^ Center of Medical Genetics, Faculty of Medicine and Health Sciences, University of Antwerp and Antwerp University Hospital, Antwerp, Belgium; ^3^ Laboratory of Physiopharmacology, University of Antwerp, Antwerp, Belgium

**Keywords:** hiPSC-CM, structural cardiotoxicity, biomarkers, miRNA, cardiotoxicity, preclinical

## Abstract

**Introduction:**

Drug-induced cardiotoxicity is one of the main causes of attrition due to safety in preclinical and clinical development; therefore, identifying novel assays and/or biomarkers to detect potentially harmful candidates is pivotal for the pharmaceutical industry. Over the past decade, microRNAs (miRNAs) have been proposed as alternative translatable biomarkers for cardiotoxicity. Although miRNAs could be useful for detection of cardiotoxicity, they are not routinely assessed in preclinical drug development.

**Methods:**

The current study aimed to investigate dysregulation of miRNAs in human-induced pluripotent stem cell-derived cardiomyocytes (hiPSC-CM) and their culture media after exposure to a set of cardiotoxic agents known to cause structural cardiotoxicity by different mechanisms of action. Dose-response analysis of intracellular miRNA expression was conducted after 72-hour incubation with 29 drugs, while the presence of miRNAs in the culture media was evaluated at 24-, 48-, and 72-hour post-treatment in response to 7 selected treatments.

**Results:**

As a result, we confirmed the upregulation of the following intracellular miRNAs across various drug classes: hsa-miR-96-5p, hsa-miR-126-3p, hsa-miR-133b, hsa-miR-146b-5p, hsa-miR-182-5p, hsa-miR-187-3p and hsa-miR-365a-5p. Interestingly, miRNAs expression in the cell culture media represented different patterns and magnitudes of upregulation, compared to the intracellular miRNAs. hsa-miR-133b, hsa-miRNA-184 and hsa-miR-208b-3p were found to be upregulated the most in the cell culture media.

**Discussion:**

The combination of intracellular and secreted miRNAs in hiPSC-CM might expand the tools for early identification of structural cardiotoxicity in preclinical drug discovery and provide a potential link to circulating miRNAs in patients with drug-induced cardiotoxicity.

## 1 Introduction

Throughout the phases of drug discovery and development, *in vitro* and *in vivo* studies are essential to identify potential hazards associated with new drug candidates and to provide a comprehensive safety profiling (Weaver and Valentin, 2019). With drug-induced cardiotoxicity remaining one of the major causes of drug attrition (Zhao et al., 2017; Wang et al., 2019), non-clinical studies need to offer a valid translational tool for de-risking novel drug candidates, as well as to help to identify adverse effects that are not detectable early in clinical trials (Seltzer et al., 2019; Guns et al., 2020). Evidence in the literature on the employment of human-induced pluripotent stem cell-derived cardiomyocytes (hiPSC-CM) models for cardiac safety evaluation is continuously increasing. Numerous publications demonstrate the ability of hiPSC-CM to predict electrophysiological drug responses and their utility in disease modelling (Burridge et al., 2015; Blinova et al., 2017; 2018; Sharma et al., 2017; Burnett et al., 2019). Moreover, assays based on hiPSC-CM are believed to replicate pivotal physiological functions of adult cardiomyocytes and may have a better predictive value as compared to cells of animal origin (Casini et al., 2017; Pourrier and Fedida, 2020). Nevertheless, hiPSC-CM have an immature phenotype in terms of morphology, contractility, and electrophysiology. Over the years, functional characterization of these cell lines has been extensively performed (Blinova et al., 2017; Kanda et al., 2018; Kussauer and Lemcke, 2019). Electrophysiological endpoints are typically analyzed by multi-electrode array (MEA) platforms, alongside other established techniques such as patch-clamp and voltage-sensitive dyes. In addition, contractility can be measured to estimate the impact on the sarcomere length and, in general, on the disturbance of cardiomyocytes’ biomechanical characteristics. However, the prediction of drug-induced structural changes (i.e., morphological damage or loss of cellular and/or subcellular components) upon chronic exposure of hiPSC-CM remains challenging (Cross et al., 2014; Palmer et al., 2020; Gryshkova et al., 2022). Considering the concerns that structural cardiotoxicity represents in the clinic, especially with chemotherapeutic agents, the identification of predictive assays/biomarkers remains an important challenge for drug development.

Over the past years, several studies have reported dysregulation of microRNAs (miRNAs) expression as a potential assay to detect cardiovascular liabilities (Romaine et al., 2015; Cortez-Dias et al., 2016; Skála et al., 2019). miRNAs are small noncoding RNAs, around 20 nucleotides in length, which regulate gene expression in several biological and physiological processes by targeting messenger RNA (mRNA). miRNAs mostly interact with the 3′ untranslated region (3′ UTR) of target mRNAs to induce mRNA degradation and repression of translation. miRNAs have been linked to different cardiovascular events such as myocardial infarction, hypertrophy, coronary artery disease, fibrosis, and heart failure (Sayed et al., 2014; Vegter et al., 2016; Viereck and Thum, 2017; Kim et al., 2018; Li et al., 2018; Ruggeri et al., 2018; Mirna et al., 2019; Skála et al., 2019). miRNAs have several characteristics that make them attractive predictive biomarkers for cardiotoxicity: 1) stability in biofluids as they can be secreted in exosomes protected from RNases; 2) detectability in biofluids which may not require invasive collection techniques; 3) tissue or disease specificity; 4) abundance; 5) early expression before substantial damage to the cells had occurred (Mikaelian et al., 2013; Koturbash et al., 2015; Skála et al., 2019). In previous work of our laboratory, Gryshkova et al. performed a broad profiling of miRNAs by Next-Generation Sequencing (NGS) in hiPSC-CM treated for 72 h with a set of cardiotoxic drugs (Gryshkova et al., 2022). These drugs were classified as functional, affecting the main functions of cardiomyocytes (9 drugs), structural, inducing alterations in the structure or the depletion of cellular and/or subcellular components in cardiomyocytes (9 drugs), or general cardiotoxicants, inducing both functional and structural changes (12 drugs). Several miRNAs were found to be upregulated in structural and general groups but not following the treatment with functional cardiotoxicants. However, these results were not confirmed in follow-up studies with a broader set of structural cardiotoxicants, nor was the concentration-response relationship evaluated.

In this study, we aimed to confirm the upregulation of miRNAs, previously associated with structural cardiotoxicity, using a hiPSC-CM model and a broad panel of structural cardiotoxicants. This assessment was combined with continuous monitoring of impedance and hiPSC-CM function. In addition, by sampling and analyzing the culture media of treated hiPSC-CM over different time points, we assessed extracellular miRNAs.

## 2 Materials and methods

### 2.1 Human-induced pluripotent stem cell-derived cardiomyocytes (hiPSC-CM)

**FIGURE 1:**
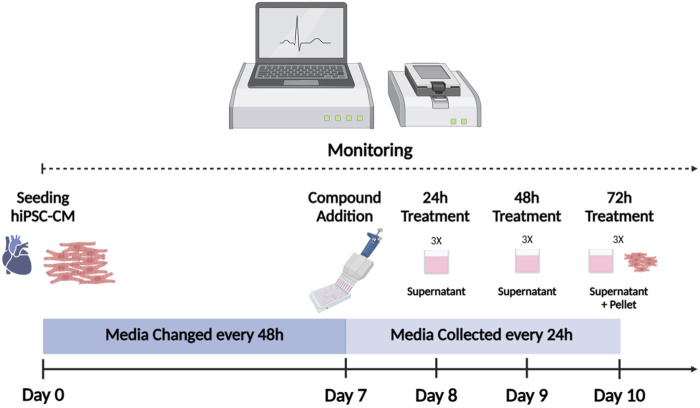
hiPSC-CM workflow. iCell^2^ were cultured for 7 days on xCELLigence ePlates before drug treatment. Baseline recording of four hours was performed for normalization before drug treatment. Following drug exposure, culture media was replaced (with the treatment) every 24 h. Culture media was preserved for miRNA analysis. Impedance and electrophysiological parameters were monitored by RTCA CardioECR throughout the whole experiment. After 72 h of treatment, hiPSC-CM were collected for miRNAs analysis. hiPSC-CM = human-induced pluripotent stem cell-derived cardiomyocytes; RTCA CardioECR = Real-Time Cell Analysis Cardio ExtraCellular Recording. (Picture created with Biorender.com)

All experiments were performed using iCell Cardiomyocytes^2^ (iCell^2^) (#01434, C1016, lot:105567/106049, FUJIFILM Cellular Dynamics, Inc. (FCDI), Madison, WI, USA), as previously described (Palmer et al., 2020; Gryshkova et al., 2022). Cryopreserved iCell^2^ hiPSC-CM were thawed according to the manufacturer’s recommendations and plated at a density of 50,000 cells/well in 48-well xCELLigence ePlates (Agilent Technologies, Inc. Santa Clara, CA, USA). To extract total RNA from the culture media, hiPSC-CM were plated on 24-well plates by seeding 275,000 cells per mL per well. hiPSC-CM were maintained for 7 days in 200 µL of iCell^2^ Maintenance Medium (iCell^2^ MM; FCDI) for the 48-well ePlate and in 1 mL for 24-well plate, before drug treatment. Culture media change was performed every 2 days. Baseline recording of four hours was performed for normalization before drug treatment. Figure 1 represents the workflow used for RTCA CardioECR and miRNA experiments.

### 2.2 Drug treatment and concentration selection

Drugs were selected according to their mechanisms of action and effect on the cardiovascular system, literature evidence of cardiotoxicity and clinical data. Multiple drug concentrations (4x) were selected based on an extensive literature search (Table 1). In general, maximum drug concentrations covered 10 times the total C_max_. For doxorubicin, seven different concentrations were initially tested to exclude highly cytotoxic concentrations; further analysis took into consideration only four concentrations (1, 0.3, 0.1, 0.03 µM) (Table 1; Supplementary Table S1, S3). Drugs were purchased from Millipore-Sigma (St Louis, MO, USA), Tocris (Abingdon, UK), MRIGlobal (Kansas City, MO, USA), and Selleckchem (Houston, TX, USA). Drug stocks were prepared in 100% dimethyl sulfoxide (DMSO, D8418, Sigma-Aldrich) at a concentration 1000 times higher than the upper drug dose tested. Stock solutions were diluted 1:1000 in iCell^2^ MM before treatment and equilibrated at 37°C. As vehicle control, DMSO 0.1% was added to the media. Drug treatments on biological replicates (n = 3) were refreshed daily, and culture media was collected for further miRNA analysis. After 72 h, cell pellets were collected and stored at −80°C until total RNA extraction.

**TABLE 1:** Drug responses in hiPSC-CM analyzed by RTCA CardioECR.

Compound class	Compound name	Cmax total (µM)	Concentration (µM)	Cell index (CI) MEC, µM (%)	Beating rate (BR) MEC, µM (%)
Anthracyclines and derivatives	Doxorubicin	1.60	0.003, 0.01, 0.1, 0.3	0.1 (−50%)	ND
	Epirubicin	16.60	0.3, 1, 3, 10	0.3 (−65%)	0.3 (+24%)
	Daunorubicin	0.31–7.84	0.1, 0.3, 1, 3	0.1 (−32%)	0.1 (+21%)
	Idarubicin	0.12–3.80	0.1, 0.3, 1, 3	0.1 (−47%)	0.1 (+34%)
	Mitoxantrone	3.42	0.1, 0.3, 1, 3	0.1 (−24%)	0.1 (+36%)
Tyrosine Kinase inhibitors (TKIs)	Sunitinib	0.18–0.25	0.01, 0.1, 0.3, 1	ND	1 (−32%)
	Erlotinib	3.15–4.80	0.23, 0.77, 2.3, 7.7	ND	0.77 (−20%)
	Nilotinib	0.84–4.27	0.01, 0.1, 0.3, 1	ND	ND
	Dasatinib	0.97	0.3, 1, 3, 10	1 (+21%)	0.3 (−36%)
	Imatinib	3.54	0.3, 1, 3, 10	ND	ND
	Lapatinib	4.18	0.1, 0.3, 1, 3	ND	**ND**
Microtubular disruptors	Vincristine	0.05	0.0003, 0.003, 0.03, 0.3	0.003 (−22%)	ND
	Vinblastine	0.035	0.0003, 0.003, 0.03, 0.3	0.3 (−24%)	0.003 (+20%)
	Vinorelbine	0.81	0.1, 0.3, 1, 3	0.1 (−23%)	1 (−20%)
	Endothelin-1	0.000002	0.0003, 0.003, 0.03, 0.3	ND	ND
	Paclitaxel	21.90	0.3, 1, 3, 10	0.3 (−27%)	3 (+20%)
Proteasome Inhibitors	Ixazomib Citrate	0.21	0.03, 0.1, 0.3, 1	0.3 (−37%)	0.03 (+33%)
	Bortezomib	0.14–2.80	0.01, 0.03, 0.1, 0.3	0.03 (−20%)	Q (at 0.1 µM)
	Carfilzomib	5.88	0.3, 1, 3, 10	0.3 (−85%)	Q (at 0.3 µM)
Effect on DNA/RNA integrity	5-FluoroUracil	4.60	0.3, 1, 3, 10	ND	ND
	Pentamidine	1.80	0.1, 0.3, 1, 3	3 (−28%)	3 (−20%)
	Etoposide	33.40	0.3, 1, 3, 10	ND	ND
	Cyclophosphamide	126.00	3, 10, 30, 100	ND	ND
Other Mechanism of Action (MoA)	Dexfenfluramine	0.80	0.01, 0.03, 0.1, 0.3	ND	ND
	Tegaserod	0.08	0.01, 0.03, 0.1, 0.3	ND	ND
	BMS-986094	N/A	0.1, 0.3, 1, 3	ND	ND
	Milrinone	0.62–1.18	0.1, 0.3, 3, 10	ND	ND
	Arsenic Trioxide	0.91–12.1	0.016, 0.048, 0.16, 0.48	ND	0.048 (+24%)
	Pergolide	12.7	0.3, 1, 3, 10	ND	ND

The table illustrates different RTCA CardioECR parameters measured in hiPSC-CM following the 72-hour treatment with several structural cardiotoxicants, grouped by drug classes. The results indicate the Minimum Effective Concentration (MEC) i.e., the lowest concentration that caused at least 20% change from baseline, and the magnitude of the effect is shown as a percentage (in brackets). Q= quiescence (beat stop); ND – not detected, magnitude of change was lower than 20%.

### 2.3 RTCA CardioECR assessment

After seeding on fibronectin-coated xCELLigence ePlates (Agilent Technologies, Inc. Santa Clara, CA, USA), iCell^2^ CM were monitored continuously through impedance electrodes. The following impedance parameters were measured: Cell Index (CI, measure of cell “health” and monolayer integrity), beating rate (BR) and amplitude (CI Amplitude, magnitude of contraction). Electrophysiology-like parameters (Field Potential Duration (FPD), considered as a surrogate for QT in *in vivo* studies, and Spike Amplitude (SA), depolarization induced by Na^+^ channel opening) were measured starting from day 4–6, following the monolayer formation. Changes in CI, BR and CA of at least 20%, compared to baseline and DMSO-treated controls, were considered biologically significant (Koci et al., 2017; Gryshkova et al., 2022). Moreover, changes ≥10% in FPD and ≥40% in SA (due to the high variability of SA parameter), were deemed biologically significant (Zhang et al., 2016; Delaunois et al., 2023). All RTCA CardioECR data were double normalized (DMSO control and the baseline) with no further statistical group comparison for the normalized parameters. The variability of the data within the treatment groups was assessed by standard deviation.

### 2.4 RNA extraction, cDNA synthesis and RT-qPCR

Total RNA was extracted from hiPSC-CM using the miRNeasy 96 Kit (Qiagen, Hilden, Germany) following the manufacturer’s recommendations. After the extraction, RNA purity was assessed by NanoDrop™ 8000 Spectrophotometer (Thermo Fisher Scientific Inc.). 5–10 ng/μL of RNA per sample were reverse transcribed and pre-amplified by using the TaqMan™ Advanced miRNA cDNA Synthesis Kit (Thermo Fisher Scientific Inc.). The synthesized cDNA was used for RT-qPCR with selected TaqMan™ assays (Thermo Fischer Scientific Inc.). RT-qPCR was carried out following the Applied Biosciences TaqMan miRNA expression assay protocol on a ViiA7 instrument (Thermo Fisher Scientific, Inc.). Two pilot experiments were performed using doxorubicin and mitoxantrone treatments in 24- and 96-well plate formats in biological triplicates with two miRNAs of interest (miR-187-3p and miR-182-5p), as single probes. We observed that the intra-well variability for these miRNAs ranged between 10-35% (CV, coefficient of variability), data not shown. Therefore, to maximize the number of drugs and concentrations, explored in the same study, we pooled the triplicates for intracellular profiling of miRNAs. Therefore, no statistical analysis was performed on the data of miRNA profiling in the pellets of hiPSC-CM.

hiPSC-CM culture media was collected daily from 24-well plates, and centrifuged (+4°C, 11,000 rpm) for 5 min to remove cellular debris/cells. Total RNA was extracted from 300 µL of supernatant by Trizol/Chloroform phase separation and purification of total RNA by miRNeasy 96 Kit (Qiagen, Hilden, Germany). The spike-in control cel-miR-39 (Qiagen, Hilden, Germany) was used to check the quality of samples and PCR efficiency. DNase treatment (RNase-Free DNase Set (Qiagen, Hilden, Germany)) was applied according to the manufacturer’s instructions. After the RNA extraction, the same RT-qPCR protocol as for hiPSC-CM lysate was followed. Multiple unpaired t-tests were performed to compare delta CT-values (CT-values normalized to the housekeeping miRNAs) of the treatment groups to the DMSO control.

## 3 Results

### 3.1 RTCA CardioECR assessment of hiPSC-CM treated with structural cardiotoxicants

Based on the previously generated data in our laboratory (Gryshkova et al., 2022), an expanded panel of 29 structural cardiotoxicants, representing different drug classes, was selected for RTCA CardioECR assessment. Specifically, the drugs tested were classified into six families: 1) anthracyclines and anthracycline-like drugs, 2) Tyrosine Kinase Inhibitors (TKIs), 3) microtubular disruptors (taxanes and vinca alkaloids), 4) proteasome inhibitors, 5) drugs with effect on DNA/RNA integrity, and 6) drugs with different mechanisms of action (MoA). We studied drug responses in hiPSC-CM by measuring impedance (CI, BR, CI Amplitude) and electrophysiology (FPD, SA) parameters during 72-hour treatment (Supplementary Table S1). The CI parameter, which reflects on morphological changes in hiPSC-CM, can be considered a relevant parameter to detect structural changes in cardiomyocytes. Therefore, we mostly focused on impedance parameters (CI, BR) to analyze the effects of structural cardiotoxicants in hiPSC-CM (Table 1) and subsequently compare the data to miRNA analysis.

Among all the drugs tested in hiPSC-CM, anthracyclines and proteasome inhibitors showed the strongest effect on CI (65% and 85% decrease, respectively). Except for doxorubicin, other drugs in these two drug classes also significantly affected the BR. In some cases, it resulted in quiescence (beat stop) already at the lowest concentration tested (i.e., epirubicin, bortezomib, carfilzomib). Among TKIs, generally, no significant effect was observed on CI or BR during 72-hour treatment, except for dasatinib. For several drugs tested (i.e., drugs with effect on DNA/RNA integrity and drugs with other MoA), the change in CI and BR did not achieve 20% and was marked as ND–not detected (Table 1). Two functional drugs, E4031 (30 nM) and nifedipine (300 nM), were used as positive controls to monitor the electrophysiological responses of hiPSC-CM. Early After Depolarizations (EADs) were observed after treatment with E4031, while an increase in BR and FPD shortening were observed after treatment with nifedipine, confirming expected drug responses in hiPSC-CM (data not shown).

### 3.2 Analysis of intracellular miRNAs upregulation in hiPSC-CM

**FIGURE 2:**
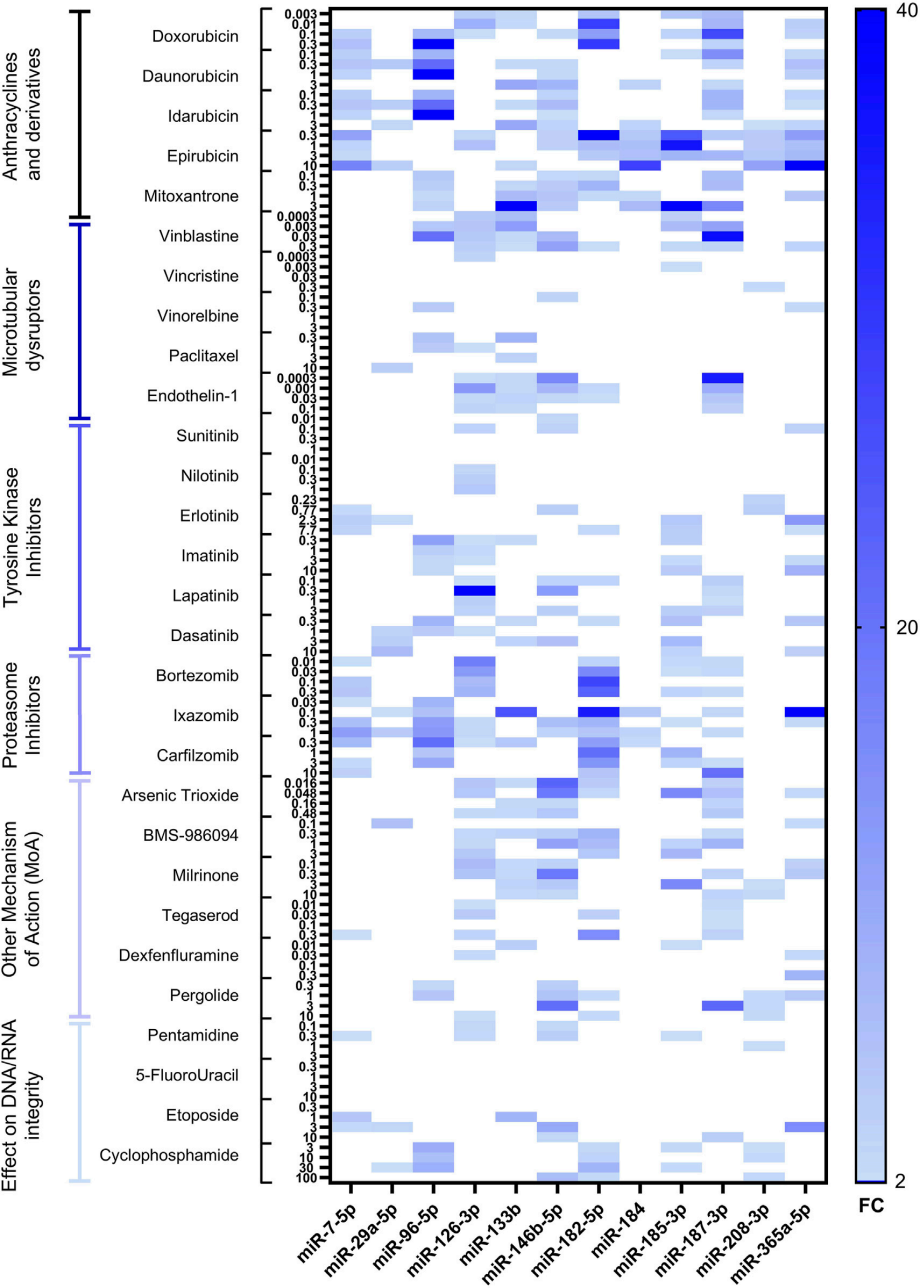
Heatmap of differentially expressed miRNAs in hiPSC-CM after 72-hour treatment with 29 structural cardiotoxicants. The heatmap shows upregulated miRNAs with fold change ≥2. miRNAs tested are indicated on the bottom of the heatmap, fold change–on the right, the treatments, divided by drug classes, at multiple concentrations–on the left.

Following RTCA CardioECR assessment, hiPSC-CM cell pellets were collected, and total RNA was extracted and purified to investigate miRNAs upregulation by RT-qPCR. Hsa-miR-125-5p, hsa-miR-191-5p, and hsa-miR-26b-5p were used as normalizers based on previously generated data (Gryshkova et al., 2022). For the initial assessment of intracellular miRNAs in 12 different treatments, encompassing various drug classes, we used a panel of 17 miRNAs, which we selected from the publicly available data focusing mainly on *in vitro* models and structural cardiotoxicants (Supplementary Table S2). Based on this initial analysis, we further excluded five miRNAs (hsa-miR-1-3p, hsa-miR-21-5p, hsa-miR-34a-5p, miR-146a-5p, hsa-miR-320a-3p), which either were not detected or showed a high variability. Therefore, 12 miRNAs (hsa-miR-7-5p, hsa-miR-29a-5p, hsa-miR-96-5p, hsa-miR-126-3p, hsa-miR-133b, hsa-miR-146b-5p, hsa-miR-182-5p, hsa-miR-184, hsa-miR-185-5p, hsa-miR-187-3p, hsa-miR-208b-3p, hsa-miR-365a-5p) were analyzed with other 17 treatments. The results of miRNA dysregulation in hiPSC-CM with a total of 29 structural cardiotoxicants are shown in Figure 2 and Supplementary Table S3.

The tested miRNAs did not change expression patterns in the DMSO control (data not shown). Among all treatment categories, anthracyclines and proteasome inhibitors were associated with the most pronounced upregulation of miRNAs. Additionally, significant miRNA overexpression was observed with the vinca alkaloid vinblastine and the monolayer disruptor endothelin-1, as well as with two agents classified under “other mechanism of action (MoA)” drugs: the phosphodiesterase inhibitor milrinone and the antineoplastic agent arsenic trioxide.

In our dataset, some miRNAs showed a very high magnitude of upregulation (exceeding fold change of ≥10); therefore, we refer to these miRNAs as highly upregulated. Seven miRNAs candidates were highly upregulated, at least once, across different treatments (hsa-miR-96-5p, hsa-miR-126-3p, hsa-miR-133b, hsa-miR-146b-5p, hsa-miR-182-5p, hsa-miR-187-3p and hsa-365a-5p). As mentioned above, anthracyclines, i.e., doxorubicin, demonstrated the highest fold change for hsa-miR-182-5p and hsa-miR-187-3p, as well as a significant upregulation of other selected miRNAs. The microtubular disruptor vinblastine induced upregulation of hsa-miR-96-5p, hsa-miR-126-3p, hsa-miR-146b-5p and hsa-miR-187-3p, while the peptide endothelin-1 induced the upregulation of hsa-miR-126-3p, hsa-miR-133b, hsa-miR-146b-5p, hsa-miR-182-5p and hsa-miR-187-3p. Milrinone treatment did not induce overexpression of hsa-miR-96-5p but showed high upregulation of hsa-miR-146b-5p (Supplementary Table S3). Proteasome inhibitor drugs, i.e., ixazomib, induced upregulation of several miRNAs, with the highly upregulated hsa-miR-133b, hsa-miR-182-5pand hsa-miR-365a-5p (Supplementary Table S3). The magnitude of miRNA upregulation for TKIs was lower compared to other drug classes. Nevertheless, erlotinib induced upregulation of three miRNAs (hsa-miR-146b-5p, hsa-miR-182-5p, hsa-miR-365a-5p), with miR-365a-5p showing the highest fold change (Supplementary Table S3). Pentamidine, an antiprotozoal agent causing inhibition of DNA/RNA synthesis and block of hERG trafficking, induced upregulation of two miRNAs (hsa-miR-126-3p and hsa-miR-146b-5p) (Supplementary Table S3). The smallest fold change was observed after treatment with the TKIs sunitinib and nilotinib, the vinca alkaloids vincristine and vinorelbine, pentamidine, and 5-fluorouracil. Overall, no clear concentration-dependent miRNAs upregulation was observed for the drugs tested. On the contrary, for some of the treatments, miRNAs upregulation showed an inverse relationship with the drug concentration–the lowest upregulation of miRNAs was detected at the highest concentration tested (i.e., doxorubicin, vinblastine, endothelin-1, milrinone, BMS-986094, arsenic trioxide).

### 3.3 Comparison of RTCA CardioECR and miRNA expression data in hiPSC-CM in response to structural cardiotoxicants

Based on the data generated in both assays, RTCA CardioECR and miRNA expression, we compared impedance parameters (CI, BR) and upregulation of miRNAs for the same treatment. For this purpose, we considered the minimum effective concentration (MEC), the lowest concentration that induced the change in CI or BR, ≥20%, and fold change ≥2 for miRNA expression. Table 2 shows the MEC values for CI and BR, as well as all miRNAs upregulated for each drug tested. In most cases, the upregulation of multiple miRNAs was detected at lower concentrations compared to the concentrations that induced changes in CI and BR. For 12 drugs tested, changes ≥20% in CI and BR were not detected, while upregulation of multiple miRNAs was observed. Overall, out of 29 drugs tested, only 5-FluoroUracil did not induce either changes in CI or BR above 20% or miRNA expression above 2-fold change.

**TABLE 2:** Comparison of RTCA CardioECR data and intracellular miRNAs expression in hiPSC-CM.

Drug/group per class		RTCA, MEC (CI, BR)	miRNAs hiPSC-CM pellet, MEC overall
Anthracyclines and derivatives	Doxorubicin	0.1 µM (CI)	0.03 µM (miR-34a-5p, miR-146b-5p, miR-187-3p)
	Epirubicin	0.3 µM (CI, BR (Q))	0.3 µM (miR-7-5p, miR-29a-5p, miR-126-3p, miR-146b-5p, miR-182-5p, miR-184, miR-185-3p, miR-187-3p, miR-208b-3p, miR-365a-5p)
	Daunorubicin	0.1 µM (CI, BR)	0.1 µM (miR-96 -5p, miR-185-3p, miR-187-3p, miR-365a-5p)
	Idarubicin	0.1 µM (CI, BR)	0.3 µM (miR-96 -5p, miR-126-3p, miR-133b, miR-185-3p)
	Mitoxantrone	0.1 µM (CI, BR)	0.1 µM (miR-96 -5p, miR-146b-5p, miR-182-5p, miR-187-3p, miR-365a-5p)
Tyrosine Kinase Inhibitors (TKIs)	Sunitinib	1 µM (BR)	0.01 µM (miR-146b-5p)
	Erlotinib	0.77 µM (BR)	0.23 µM (miR-1-3p, miR-21-5p, miR-34a-5p, miR-208b-3p)
	Nilotinib	ND	0.3 µM (miR-126-3p)
	Dasatinib	1 µM (CI), 0.3 µM (BR)	0.3 µM (miR-96-5p, miR-133b, miR-182-5p, miR-185-3p, miR-365a-5p)
	Imatinib	ND	0.3 µM (miR-96-5p, miR-126-3p, miR-133b, miR-185-3p)
	Lapatinib	ND	0.1 µM (miR-126-3p, miR-146b-5p, miR-182-5p, miR-187-3p)
Microtubular disruptors	Vincristine	0.003 µM (CI)	0.0003 µM (miR-126-3p)
	Vinblastine	0.3 µM (CI), 0.003 µM (BR)	0.0003 µM (miR-126-3p, miR-133b, miR-185-3p)
	Vinorelbine	0.1 µM (CI), 1 µM (BR)	0.1 µM (miR-34a-5p, miR-146b-5p)
	Endothelin-1	ND	0.0003 µM (miR-34a-5p, miR-126-3p, miR-133b, miR-146b-5p, miR-187-3p, miR-320a-5p)
	Paclitaxel	0.3 µM (CI), 3 µM (BR)	0.3 µM (miR-96-5p, miR-133b)
Proteasome Inhibitors	Ixazomib Citrate	0.3 µM (CI), 0.03 µM (BR)	0.03 µM (miR-126-3p, miR-29a-5p, miR-7-5p, miR-96-5p)
	Bortezomib	0.03 µM (CI), Q (BR)	0.01 µM (miR-126-3p, miR-182-5p, miR-185-3p, miR-187-3p, miR-7-5p)
	Carfilzomib	0.3 µM (CI, BR (Q))	0.3 µM (miR-126-3p, miR-133b, miR-182-5p, miR-184, miR-187-3p, miR-7-5p, miR-96-5p)
Effect on DNA/RNA integrity	5-FluoroUracil	ND	ND
	Pentamidine	3 µM (CI, BR)	0.1 µM (miR-126-3p, miR-146b-5p, miR-208b-3p)
	Etoposide	ND	0.1 µM (miR-187-3p)
	Cyclophosphamide	ND	3 µM (96-5p, 182-5p, 185-3p, 208b-3p)
Other Mechanism of Action (MoA)	Dexfenfluramine	ND	0.01 µM (miR-133b, miR-185-3p)
	Pergolide	ND	0.3 µM (miR-96-5p, miR-146b-5p)
	Tegaserod	ND	0.01 µM (miR-126-3p, miR-187-3p)
	BMS-986094	ND, 0.048 µM (BR)	0.1 µM (miR-29a-5p, miR-365a-5p)
	Milrinone	ND	0.1 µM (miR-126-3p, miR-133b, miR-146b-5p)
	Arsenic Trioxide	ND	0.016 µM (miR-126-3p, miR-133b, miR-146b-5p, miR-182-5p, miR-187-3p)

Minimum effective concentration (MEC) is listed for CI and BR; for miRNAs – the lowest concentration tested which produced miRNA upregulation (fold change ≥2), in most cases upregulation of several miRNAs. CI = Cell Index; BR = Beat Rate; ND – not detected, magnitude of change was lower than 20% for CI/BR, and fold change <2 for miRNAs.

### 3.4 Analysis of miRNAs upregulation in the culture media of hiPSC-CM treated with structural cardiotoxicants

Analysis of miRNA in the culture media of hiPSC-CM treated with structural cardiotoxicants was performed at 24-, 48- and 72-hour post-treatment. Considering the high number of experimental conditions tested in hiPSC-CM and the low throughput of the assay, we selected one concentration per drug for seven treatments (doxorubicin, vinblastine, vincristine, arsenic trioxide, milrinone, sunitinib, erlotinib). This selection was guided by the intracellular miRNA profiles in the hiPSC-CM lysates, aiming to analyze treatments spanning various drug classes. The treatment concentrations were selected based on the MEC for CI to ensure that the preliminary evidence of cardiotoxicity in hiPSC-CMs could potentially lead to miRNA release in the culture media. For drugs where an effect on CI was not detected, we selected the highest tested concentration, provided it was in the range of 10x therapeutic Cmax. For vincristine, where MEC was at a lower concentration, we prioritized using the highest tested concentration to maximize miRNA detection in the supernatant as high variability was observed in preliminary studies.

**FIGURE 3:**
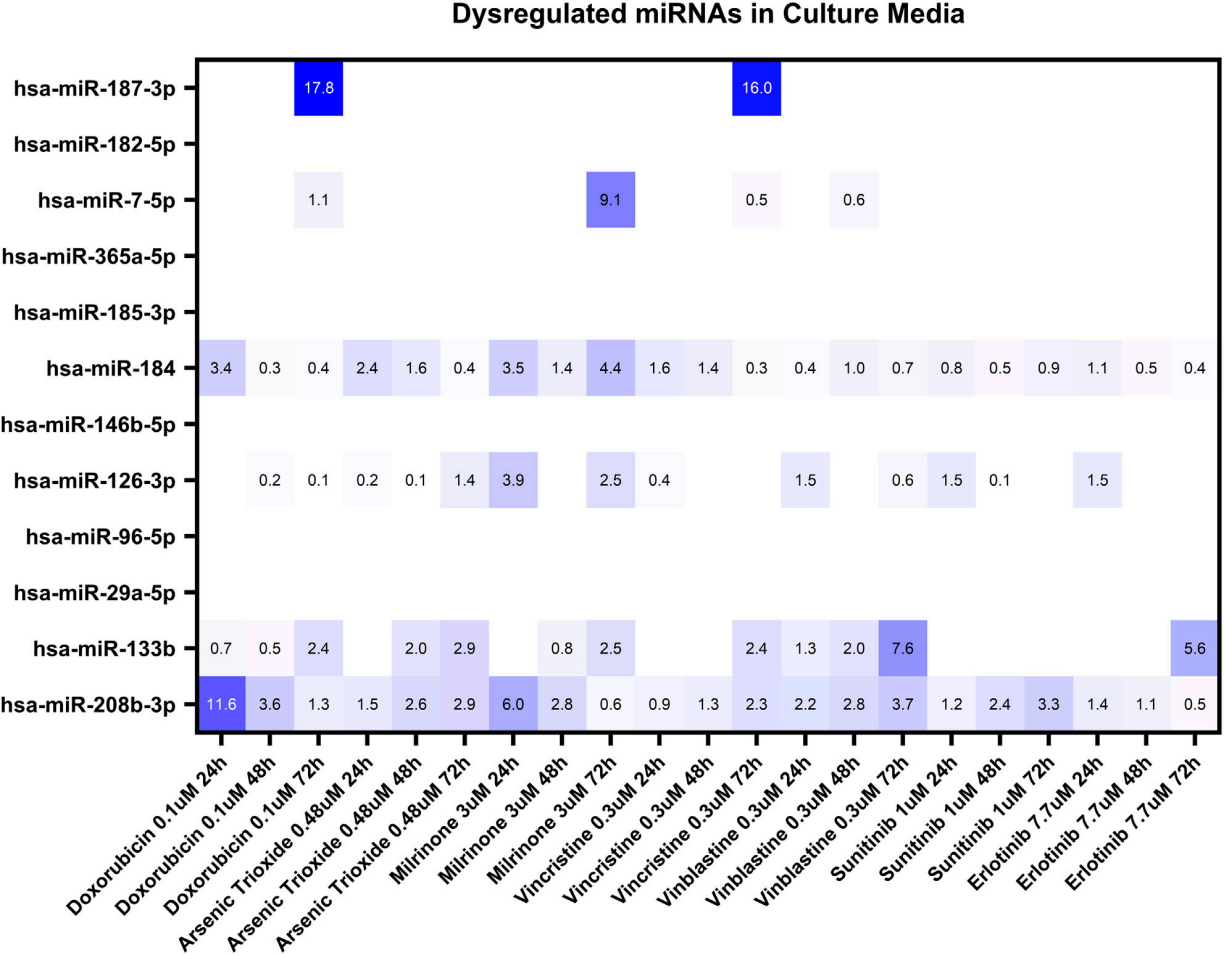
Dysregulation of twelve miRNAs in the culture media of hiPSC-CMs treated with seven structural cardiotoxicants (doxorubicin 0.1 μM, arsenic trioxide 0.48 μM, milrinone 3 μM, vincristine 0.3 μM, vinblastine 0.3 μM, sunitinib 1 μM, and erlotinib 7.7 μM). The analysis was conducted at 24-, 48-, and 72-hour post-treatment. Fold changes (double normalization to housekeeping miRNAs and DMSO/vehicle control) ≥ 2 are considered biologically meaningful.

Based on our miRNA profiling in hiPSC-CM and existing literature linking miRNAs to cardiovascular events, the same panel of 12 miRNAs (hsa-miR-7-5p, hsa-miR-29a-5p, hsa-miR-96-5p, hsa-miR-126-3p, hsa-miR-133b, hsa-miR-146b-5p, hsa-miR-182-5p, hsa-miR-184, hsa-miR-185-3p, hsa-miR-187-3p, hsa-miR-208b-3p and hsa-miR-365a-5p) was used to investigate secreted miRNAs. Figure 3 shows a heat map of miRNA expression patterns in the hiPSC-CM supernatants across treatments.

**FIGURE 4:**
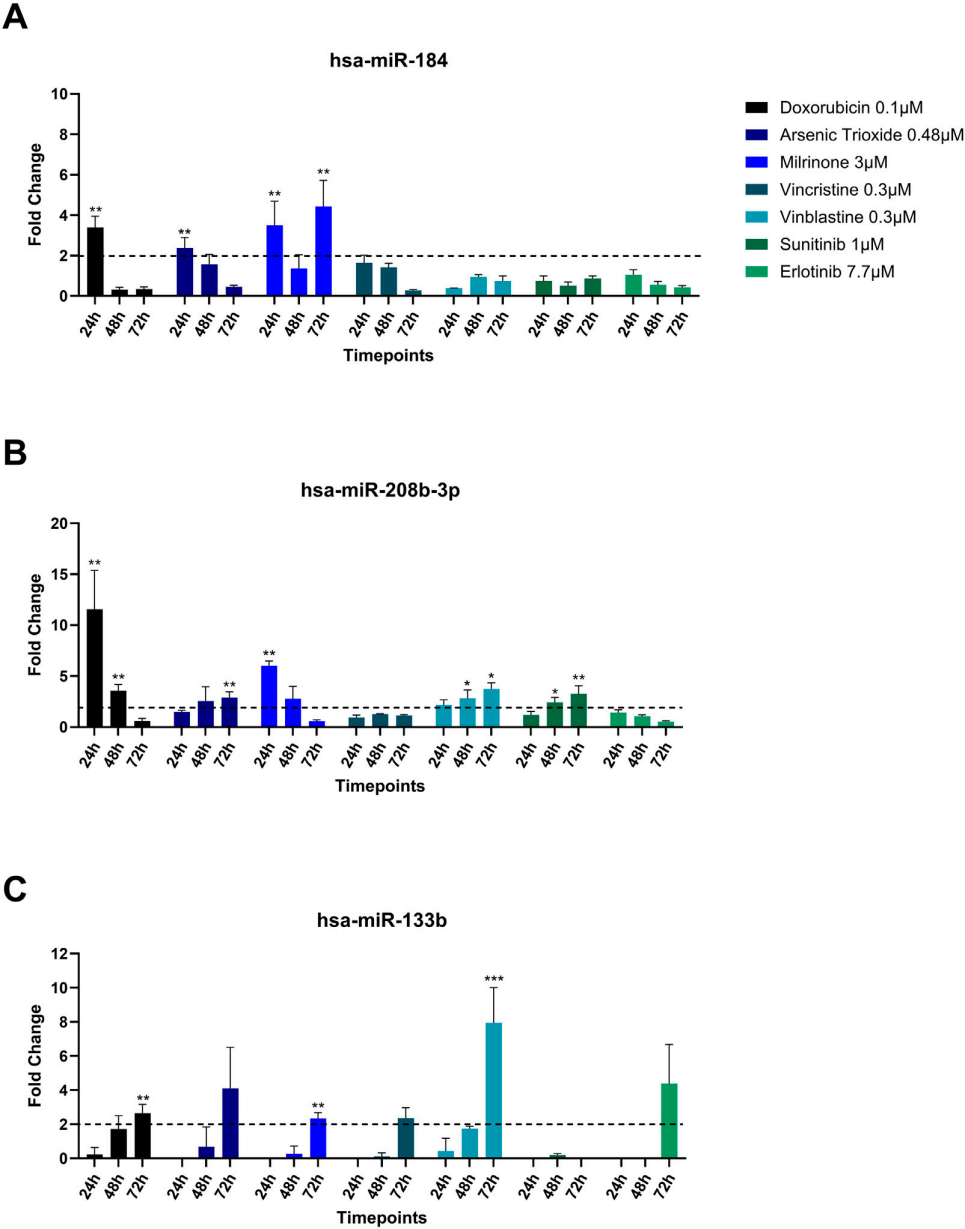
Upregulation **(A)** hsa-miR-184, **(B)** hsa-miR-208b-3p, and **(C)** hsa-miR-133b in the cell culture media of hiPSC-CM treated with structural cardiotoxicants. The dotted line represents a threshold of fold change = 2. (n = 3; *p < 0.05; **p < 0.01; ***p < 0.001; ND = Not Detected).

Among the miRNAs analyzed, hsa-miR-133b, hsa-miR-184, and hsa-miR-208b-3p demonstrated significant upregulation (fold change≥ 2) in the culture media in response to multiple treatments (Figure 4). hsa-miR-184 was upregulated following 24-hour exposure to doxorubicin, arsenic trioxide, and milrinone. However, at 48-hour post-treatment its levels decreased and only for milrinone, upregulation was detected again at 72-hour time point (Figure 4A).

hsa-miR-208b-3p expression levels declined over time in response to doxorubicin and milrinone, while, following treatment with vinblastine and sunitinib, an opposite trend was observed (Figure 4B). For hsa-miR-133b, upregulation was detected only after 72 h of treatment with doxorubicin, vinblastine, and milrinone (Figure 4C).

hsa-miR-187-3p, which exhibited high upregulation levels in hiPSC-CMs across treatments, was detected in the culture media only following 72-hour exposure to doxorubicin and vinblastine (Table 3). Additionally, two miRNAs, hsa-miR-7-5p and hsa-miR-126-3p, were upregulated after 72-hour exposure to milrinone; for hsa-miR-126-3p, upregulation was also observed after 24-hour treatment. In contrast, other highly upregulated intracellular miRNAs in hiPSC-CMs, i.e., hsa-miR-96-5p and hsa-miR-182-5p, were undetectable in the culture media across all drug treatments tested (Table 3).

**TABLE 3:** Comparison between miRNAs upregulation in culture media and in hiPSC-CM.

miRNAs	Timepoint	Doxorubicin		Arsenic		Milrinone		Vincristine		Vinblastine		Sunitinib		Erlotinib	
		0.1 µM		0.48 µM		3 µM		0.3 µM		0.3 µM		1 µM		7.7 µM	
		Culture media	Intra-cellular	Culture media	Intra-cellular	Culture media	Intra-cellular	Culture media	Intra-cellular	Culture media	Intra-cellular	Culture media	Intra-cellular	Culture media	Intra-cellular
hsa-miR-187-3p	24h	ND		ND		ND		ND		ND		ND		ND	
	48h	ND		ND		ND		ND		ND		ND		ND	
	72h	17.8	27.2	ND	5.0	ND	1.2	16.0	0.7	ND	3.1	ND	0.4	ND	1.1
hsa-miR-182-5p	24h	ND		ND		ND		ND		ND		ND		ND	
	48h	ND		ND		ND		ND		ND		ND		ND	
	72h	ND	12.4	ND	2.0	ND	0.9	ND	0.4	ND	2.1	ND	0.7	ND	2.6
hsa-miR-7-5p	24h	ND		ND		ND		ND		ND		ND		ND	
	48h	ND		ND		ND		ND		0.6		ND		ND	
	72h	1.1	4.0	ND	0.6	9.1	0.5	0.5	1.0	ND	0.7	ND	1.2	ND	3.6
hsa-miR-365a-5p	24h	ND		ND		ND		ND		ND		ND		ND	
	48h	ND		ND		ND		ND		ND		ND		ND	
	72h	ND	3.6	ND	1.6	ND	0.4	ND	ND	ND	3.3	ND	0.8	ND	2.1
hsa-miR-185-3p	24h	ND		ND		ND		ND		ND		ND		ND	
	48h	ND		ND		ND		ND		ND		ND		ND	
	72h	ND	3.4	ND	ND	ND	16.3	ND	1.1	ND	2.7	ND	0.2	ND	4.6
hsa-miR-184	24h	3.4		2.4		3.5		1.6		0.4		0.8		1.1	
	48h	0.3		1.6		1.4		1.4		1.0		0.5		0.5	
	72h	0.4	ND	0.4	ND	4.4	ND	0.3	ND	0.7	ND	0.9	ND	0.4	ND
hsa-miR-146b-5p	24h	ND		ND		ND		ND		ND		ND		ND	
	48h	ND		ND		ND		ND		ND		ND		ND	
	72h	ND	2.5	ND	5.5	ND	5.0	ND	0.3	ND	11.8	ND	0.7	ND	0.2
hsa-miR-126-3p	24h	ND		0.2		3.9		0.4		1.5		1.5		1.5	
	48h	0.2		0.1		ND		ND		ND		0.1		ND	
	72h	0.1	2.0	1.4	2.5	2.5	0.4	ND	1.4	0.6	4.3	ND	1.9	ND	0.9
hsa-miR-96-5p	24h	ND		ND		ND		ND		ND		ND		ND	
	48h	ND		ND		ND		ND		ND		ND		ND	
	72h	ND	8.1	ND	ND	ND	ND	ND	0.8	ND	ND	ND	ND	ND	0.5
hsa-miR-29a-5p	24h	ND		ND		ND		ND		ND		ND		ND	
	48h	ND		ND		ND		ND		ND		ND		ND	
	72h	ND	0.7	ND	0.3	ND	ND	ND	1.0	ND	0.2	ND	0.7	ND	1.0
hsa-miR-133b	24h	0.7		ND		ND		ND		1.3		ND		ND	
	48h	0.5		2.0		0.8		ND		2.0		ND		ND	
	72h	2.4	0.3	2.9	2.3	2.5	3.5	2.4	0.3	7.6	2.1	ND	0.2	5.6	0.7
hsa-miR-208b-3p	24h	11.6		1.5		6.0		0.9		2.2		1.2		1.4	
	48h	3.6		2.6		2.8		1.3		2.8		2.4		1.1	
	72h	1.3	0.8	2	2.0	0.6	2.1	2.3	2.2	3.7	1.1	3.3	1.7	0.5	1.0

Fold Changes (double normalization to housekeeping miRNAs and DMSO/vehicle control) ≥ 2 are considered biologically significant.

## 4 Discussion

Current *in vitro* approaches to detect cardiotoxicity, induced by drug candidates, mostly focus on the interference of a test substance with ion channels and do not include drug-induced structural cardiotoxicity (Fermini and Fossa, 2003; Brown, 2005; Blinova et al., 2018; Weaver and Valentin, 2019). Considering the concerns that structural cardiotoxicity represents in the clinic, especially with chemotherapeutic agents, the identification of predictive assays/biomarkers *in vitro* remains an important challenge for drug discovery.

Despite substantial progress in the investigation of miRNAs in cardiotoxicity, their utility as biomarkers in preclinical drug development remains limited. This could be related to insufficient validation efforts across the industry and a lack of confidence in miRNA-based assays. In this study, we confirmed the upregulation of several miRNAs, previously identified in hiPSC-CM models of cardiotoxicity, using a broader panel of cardiotoxicants. In parallel, we evaluated dose responses to the drugs in hiPSC-CM by measuring impedance and functional parameters.

We detected the upregulation of several intracellular miRNAs across various drug classes: hsa-miR-96-5p, hsa-miR-126-3p, hsa-miR-133b, hsa-miR-146b-5p, hsa-miR-182-5p, hsa-miR-187-3p and hsa-miR-365a-5p. For several drugs tested (i.e., “effect on DNA/RNA synthesis”, “other MoA”, and some of TKIs), miRNAs upregulation was observed in absence of significant changes in impedance parameters. The initial panel of 17 miRNAs was narrowed down to 12 miRNAs. For hsa-miR-1-3p, hsa-miR-21-5p, hsa-miR-34a-5p, miR-146a-5p and hsa-miR-320a-3p we could not confirm expression/upregulation in our hiPSC-CM model. This discrepancy may be explained by the difference in models used in other published studies as well as challenges linked to the detection and quantification of miRNAs.

Although several miRNAs showed differential expression in response to drug treatment, most did not exhibit a clear dose-dependent trend across four concentrations tested. The lack of a straightforward concentration-response relationship may reflect the complex nature of miRNA regulation under cardiotoxic stress. In addition to biological variability, saturation of cellular uptake mechanisms or drug targets at higher doses could result in plateaued or attenuated responses. Furthermore, miRNA expression is often subject to intricate feedback regulation—both at the transcriptional and post-transcriptional levels, which can modulate responses in a non-linear fashion. These findings underscore the importance of considering systems-level regulation when interpreting miRNA responses to varying drug concentrations.

Most of the confirmed miRNAs in hiPSC-CM were found to be associated either with apoptotic pathways and/or various cardiovascular events. The profound and sustainable upregulation of miRNAs was observed in hiPSC-CM following anthracycline treatment. Notably, hsa-miR-182-5p and hsa-miR-187-3p exhibited significant upregulation in response to doxorubicin, consistent with previously reported data (Chaudhari et al., 2016; Gryshkova et al., 2022). miR-146b-5p was shown to have cardioprotective effects by attenuating apoptotic events in a rat model of ischemic injury (Di et al., 2017), as well as by inhibiting vascular smooth muscle cell proliferation and migration, thereby mitigating atherosclerosis (Sun et al., 2020). Similarly, the overexpression of miR-133b attenuated apoptosis in a murine cardiomyocyte model following doxorubicin treatment (Li et al., 2021). miR-126-3p, which was primarily associated with endothelial dysfunction *in vitro*, in clinical studies was found to be a promising prognostic biomarker for major cardiovascular adverse events, coronary artery disease, and stroke (Yang et al., 2017; Jordan et al., 2021; Martinez-Arroyo et al., 2023). Upregulation of miR-96-5p was documented in murine heart tissues following myocardial infarction (Wang et al., 2021). The expression of miR-365a-5p is associated with hypertrophic events both *in vitro* and *in vivo*, as well as in the serum of hypertensive patients with left ventricular hypertrophy (Burridge et al., 2015; Wu et al., 2017).

miRNAs, identified in hiPSC-CM, could serve as a tool for early derisking of structural cardiotoxicity. However, the potential of these miRNAs as translatable biomarkers might be limited due to difficulties in the assessment of tissular miRNAs in the clinic. Therefore, along with the assessment of intracellular miRNAs expression in hiPSC-CM, we gained insights into the secreted miRNAs or miRNAs that could be detected in the culture media, which was not reported previously in hiPSC-CM models. By assessing selected treatments in hiPSC-CM, we demonstrated the upregulation of the following miRNAs in the culture media: hsa-miR-133b, hsa-miR-184 and hsa-miR-208b-3p.

hsa-miR-184 was significantly upregulated in the supernatant of hiPSC-CMs following treatments with doxorubicin, arsenic trioxide, and milrinone for 24 h. At later time point (72 h), miR-184 expression was no longer elevated in the supernatant in response to doxorubicin or arsenic trioxide but to milrinone. Interestingly, intracellular hsa-miR-184 was not detected in hiPSC-CM in response to the same treatments at 72 h. As we only assessed intracellular miRNAs at the 72-hour post-treatment time point, it remains unclear whether miR-184 expression could be quantified at earlier time points (i.e., 24 h and 48 h). Presence of hsa-miR-184 in the culture media only at early time point (24 h) may suggest a rapid release in response to cardiotoxic insult and a subsequent degradation in the extracellular environment. However, for milrinone, hsa-miR-184 was upregulated in the supernatant at 72 h. The mechanisms of the miRNAs release under these conditions remain unknown and highlight the complexity of miRNA regulation and release.

Preclinical studies indicate that miR-184 may predict cardiac damage by modulating apoptosis and inflammatory pathways, contributing to myocardial injury. Wang et al., 2015 demonstrated that miR-184 regulated apoptosis in response to oxidative stress in the rat heart cell line H9c2 and mouse models. Circulating miR-184 was proposed as a potential biomarker of cardiac injury in patients with Anderson–Fabry disease (AFD), which is a rare lysosomal storage disorder (Salamon et al., 2021). In a study by Jiang et al., 2022, hsa-miR-184 was found to be significantly upregulated in serum exosomes in ischemic stroke patients compared to healthy controls.

The expression of hsa-miR-208b-3p was upregulated in the culture media following the 24-hour treatment with doxorubicin and milrinone, with a subsequent decline over time. On the contrary, in response to arsenic trioxide, vinblastine, and sunitinib, the expression of hsa-miR-208b-3p exhibited a time-dependent increase. It is worth noting that intracellular miR-208-3p was upregulated in hiPSC-CM after 72-hour treatment in response to arsenic trioxide, milrinone and vincristine, which further highlights the dynamic miRNA regulation in the cell in response to insults triggered by different molecular mechanisms. miR-208b was found to be upregulated in serum and infarcted heart tissues of patients with MI (Boštjančič et al., 2010; Bronze-Da-Rocha, 2014), as well as in other cardiovascular diseases, i.e., coronary artery disease and acute coronary syndrome as well as *in vivo* models of cardiotoxicity (Widera et al., 2011; Sayed et al., 2014; Li et al., 2015; Nishimura et al., 2015; Cortez-Dias et al., 2016; Glineur et al., 2016). miR-208b-3p was observed to promote fibrosis through TGF-β1/Smad3 signalling pathway activation, and it was correlated to the release of cardiac troponins after necrosis of the cardiac tissue (Widera et al., 2011; Zhang et al., 2021). Furthermore, a study by Wang et al., 2022 showed that miR-208b-3p expression was upregulated in response to hypoxia/reoxygenation injury. The authors suggested that miR-208b-3p played a protective role against apoptosis in cardiomyocytes under stress conditions.

In our study, the expression profile of hsa-miR-133b in the culture media of hiPSC-CMs differed from that of miR-184 and miR-208b-3p. A significant upregulation was observed only after 72-hour treatment with doxorubicin, milrinone, and vinblastine, whereas treatment with arsenic trioxide, vincristine, and erlotinib resulted in an increase that was not statistically significant. Similarly to culture media, intracellular hsa-miR-133 expression was upregulated in response to milrinone, and vinblastine.

In previous studies, hsa-miR-133b was detected in *in vivo* rodent models of fibrosis and myocardial infarction (MI) after chemotherapeutic treatment, where it was found upregulated in plasma samples and extracted tissues (Nishimura et al., 2015; Hanousková et al., 2019; Li et al., 2021). It was also demonstrated that miR-133b-5p contributes to hypoxic preconditioning-mediated cardioprotection by inhibiting the activation of caspase-8 and caspase-3 apoptotic signalling, thereby reducing cardiomyocyte apoptosis following ischemia/reperfusion injury *in vivo* (Pan et al., 2018). In humans, miR-133b was shown to be diversely dysregulated according to specific cardiac pathologies. In patients with dilated cardiomyopathy, miR-133b was observed to be downregulated, both in serum samples and in explanted heart tissues (Esmaeili-bandboni et al., 2018; de Araújo Melo et al., 2019; Marinas et al., 2020; Hailu et al., 2023). On the contrary, in acute myocardial infarction, miR-133b was mainly upregulated alongside other cardiac-specific miRNAs: miR-1, miR-133a, and miR-208b (Widera et al., 2011; Sayed et al., 2014; Cortez-Dias et al., 2016; Viereck and Thum, 2017). Given that this research encompassed various drugs with pro-apoptotic and cytotoxic effects, it is plausible to anticipate the upregulation of miR-133b, miR-184 and miR-208b in hiPSC-CM and either their secretion into the media or release by damaged cells.

Despite the confirmation of the upregulation of intracellular miRNAs in response to cardiotoxicants, the analysis of hiPSC-CM secreted miRNAs presented important challenges. These limitations included a low abundance of miRNAs and insufficient sensitivity of detection and quantification methods for miRNAs in culture media as well as the lack of standardized high-throughput multiplex assays. In addition, a very high number of experimental conditions did not allow a direct comparison between all time points used for secreted and intracellular miRNAs.

A better understanding of the pathophysiological mechanisms underlying the release of these miRNAs and their potential link to the onset of cardiovascular liabilities would be important in confirming their value as translatable biomarkers. Time-dependent localization studies of the miRNAs in hiPSC-CM and/or exosomes will contribute to the development of novel assays for the early detection of structural cardiotoxicity *in vitro*. The exploration of these miRNAs in patients with drug-induced cardiotoxicity represents an important future direction for research in this field. However, the complexities inherent in such studies must be carefully considered. Many drugs in our panel are administered to oncology patients who often receive multiple medications. Moreover, cancer itself may serve as a confounding factor regarding the presence of miRNAs in serum; and there is a considerable inter-patient variability in cardiotoxicity severity in response to the same treatment. While we acknowledge that plasma miRNA profiling holds promise for future translational applications, we believe that the identified combinations of miRNAs will be most valuable in early drug discovery.

Our dataset further reflects on the complexity of miRNA expression and regulation under conditions of cardiac damage, highlighting that the predictive value of a single miRNA is insufficient to accurately assess the cardiotoxic potential of drug candidates. On top of that, the hiPSC-CM model does not replicate the intrinsic complexity of *in vivo* miRNA regulation in the heart; therefore, the limited data reported in patients might differ from the miRNA profiles seen in stem cell-derived cardiomyocytes. Nevertheless, for drug discovery and development, our findings present valuable knowledge on the potential use of miRNA-based assays in hiPSC-CM for the detection of harmful molecules and/or support of mechanistic studies.

In conclusion, we confirmed upregulation of several miRNAs (hsa-miR-96-5p, hsa-miR-126-3p, hsa-miR-133b, hsa-miR-146b-5p, hsa-miR-182-5p, hsa-miR-187-3p and hsa-365a-5p) in hiPSC-CM across different drug classes known to cause structural damage to cardiomyocytes. In addition, we generated novel data on the upregulation of hsa-miR-133b, hsa-miR-184, and hsa-miR-208b-3p in hiPSC-CM culture media. To the best of our knowledge, this study is the first attempt to employ a medium-throughput approach to detect miRNAs in the culture media of stem cell-derived cardiomyocytes subjected to the cardiac insults. In the context of drug discovery, the panels of identified miRNAs have the potential to contribute to the prediction and/or characterization of structural cardiotoxicity. When combined with other assays, these miRNAs may help refine the safety profiles of drug candidates and reduce the risk of late-stage attrition due to cardiotoxicity.

## Data Availability

The original contributions presented in the study are included in the article/Supplementary Material, further inquiries can be directed to the corresponding author.
