# Supplementary material for: Bridging the gaps in detection of structural cardiotoxicity in stem cell-derived cardiomyocytes: promise of miR-133b, miR-184 and miR-208b-3p
Source: Front Pharmacol. 2025 Jul 25;16:1584734. doi: 10.3389/fphar.2025.1584734 (PMC12332266; doi:10.3389/fphar.2025.1584734)
Supplement: Supplementary file 1 [file DataSheet1.docx]

|  | **Compound name** | **Cmax total (µM)** | **Concentration (µM)** | **Cell Index** | **Contractility Amplitude** | **Beating Rate** | **Field Potential Duration** | **Spike Amplitude (SA)** |
| --- | --- | --- | --- | --- | --- | --- | --- | --- |
| **Anthracyclines and derivatives** | Doxorubicin | 1.6 | 3, 1, 0.3 ,0.1, 0.03, 0.01, 0.003 | 0.1µM (-50%) | 0.1µM (-43%) | ND | ND | 0.01µM (-24%) |
|  | Epirubicin | 16.6 | 10, 3, 1, 0.3 | 0.3µM (-65%) | 0.3µM (-87%) | 0.3µM (+24%) | 0.3µM (-66%) | 0.3µM (-92%) |
|  | Daunorubicin | 0.310-7.84 | 3, 1, 0.3, 0.1 | 0.1µM (-32%) | 0.3µM (-82%) | 0.1µM (+21%) | 0.1µM (-50%) | 0.1µM (-93%) |
|  | Idarubicin | 0.12–3.8 | 3, 1, 0.3, 0.1 | 0.1µM (-47%) | 1µM (-43%) | 0.1µM (+34%) | 0.1µM (-39%) | 0.1µM (+38%) |
|  | Mitoxantrone | 3.42 | 3, 1, 0.3, 0.1 | 0.1µM (-24%) | 0.1µM (-29%) | 0.1µM (+36%) | 0.1µM (-27%) | 0.1µM (-53%) |
| **Tyrosine Kinase inHibitors (TKIs)** | Sunitinib | 0.181-0.25 | 1, 0.3, 0.1, 0.01 | ND | ND | 1µM (-32%) | ND | 0.01µM (-47%) |
|  | Erlotinib | 3.15-4.8 | 7.7, 2.3, 0.77, 0.23 | ND | ND | 0.77µM (-20%) | ND | 0.23µM (-39%) |
|  | Nilotinib | 0.84-4.27 | 1, 0.3, 0.1, 0.01 | ND | 0.1µM (-25%) | ND | ND | 0.01µM (+24%) |
|  | Dasatinib | 0.97 | 10, 3, 1, 0.3 | 1µM (+21%) | 10µM (-25%) | 0.3µM (-36%) | ND | 0.3µM (+20%) |
|  | Imatinib | 3.54 | 10, 3, 1, 0.3 | ND | ND | ND | ND | 0.3µM (+28%) |
|  | Lapatinib | 4.18 | 3, 1, 0.3, 0.1 | ND | ND | ND | 0.1µM (+101%) | 0.1µM (-30%) |
| **Microtubular disruptors** | Vincristine | 0.05 | 0.3, 0.03, 0.003, 0.0003 | 0.003µM (-22%) | 0.003µM (-41%) | ND | ND | 0.003µM (-46%) |
|  | Vinblastine | 0.035 | 0.3, 0.03, 0.003, 0.0003 | 0.3µM (-24%) | 0.0003µM (-21%) | 0.003µM (+20%) | ND | 0.0003µM (-24%) |
|  | Vinorelbine | 0.811 | 3, 1, 0.3, 0.1 | 0.1µM (-23%) | 0.1µM (-27%) | 1µM (-20%) | 1µM (-30%) | 0.1µM (-69%) |
|  | Endothelin-1 | 0.000002 | 0.1, 0.03, 0.001, 0.0003 | ND | 0.001µM (-20%) | ND | 0.001µM (+20%) | 0.0003µM (-52%) |
|  | Paclitaxel | 21.9 | 10, 3, 1, 0.3 | 0.3µM (-27%) | 0.3µM (-43%) | 3µM (+20%) | 3µM (+26%) | 3µM (-39%) |
| **Proteasome**  **Inhibitors** | Ixazomib Citrate | 0.213 | 1, 0.3, 0.1, 0.03 | 0.3M (-37%) | 0.1µM (-94%) | 0.03µM (+33%) | 0.03µM (-35%) | 0.03µM (+75%) |
|  | Bortezomib | 0.14-2.8 | 0.3, 0.1, 0.03, 0.01 | 0.03µM (-20%) | Q (at 0.1µM) | Q (at 0.1µM) | Q (at 0.1µM) | Q (at 0.1µM) |
|  | Carfilzomib | 5.88 | 10, 3, 1, 0.3 | 0.3µM (-85%) | Q (at 0.3µM) | Q (at 0.3µM) | Q (at 0.3µM) | Q (at 0.3µM) |
| **Effect on DNA/RNA** | 5-FluoroUracil | 4.6 | 10, 3, 1, 0.3 | ND | ND | ND | ND | ND |
|  | Pentamidine | 1.8 | 3, 1, 0.3, 0.1 | 3µM (-28%) | 0.1µM (-23%) | 3µM (-20%) | 1µM (+37%) | 0.1µM (-49%) |
|  | Etoposide | 33.4 | 10, 3, 1, 0.3 | ND | ND | ND | 3µM (-23%) | 0.3µM (+24%) |
|  | Cyclophosphamide | 126 | 100, 30, 10, 3 | ND | ND | ND | ND | ND |
| **Other Mechanism of Action (MoA)** | Dexfenfluramine | 0.8 | 0.3, 0.1, 0.03, 0.01 | ND | ND | ND | ND | 0.01µM (+51%) |
|  | Tegaserod | 0.08 | 0.3, 0.1, 0.03, 0.01 | ND | ND | ND | 0.1µM (+50%) | 0.01µM (+27%) |
|  | BMS-986094 | N/A | 3, 1, 0.3, 0.1 | ND | ND | 0.048µM (+24%) | ND | 0.16µM (-36%) |
|  | Milrinone | 0.62-1.18 | 10, 3, 0.3, 0.1 | ND | ND | ND | 0.3µM (-21%) | 0.1µM (+41%) |
|  | Arsenic Trioxide | 0.91-12.1 | 0.48, 0.16, 0.048, 0.016 | ND | ND | ND | ND | 0.016µM (+59%) |
|  | Pergolide | 12.7 | 10, 3, 1, 0.3 | ND | 0.3nM (-23%) | ND | ND | 10 µM (-32%) |

**Supplementary Table 1:** **Structural cardiotoxicants tested on RTCA CardioECR instrument**. The table illustrates different RTCA CardioECR parameters measured in hiPSC-CM cultures following the 72-hour treatment with several structural cardiotoxicants, divided by drug classes. The results indicate the Minimum Effective Concentration (MEC) i.e., the lowest concentration that caused changes from baseline and DMSO-treated controls by at least 20% (CI, BR, CA), 10% (FPD), and 40% (SA), and the magnitude of the effect, in percentage, - in brackets*.*

*Q= quiescence; ND= non-detected.*

**Supplementary Table 2.** Selection of miRNAs for assessment in hiPSC-CM treated with structural cardiotoxicants.

| **miRNAs selected for initial study of 12 treatments** | **Model** | **The direction of dysregulation** | **Reference** | **Selected for follow up studies?** |
| --- | --- | --- | --- | --- |
| hsa-miR-1-3p | Heart Tissue; Rat and mouse Cardiomyocytes; Plasma | Down; Down; Up | Boštjančič et al. 2010; Wu et al 2018; Rigaud et al 2016 | No |
| hsa-miR-7-5p | Rat ventricular cells (H9C2); Plasma | Up; Up | Li et al. 2014; Kaneto et al. 2017 | Yes |
| hsa-miR-21-5p | Mouse heart tissue and rat ventricular cells (H9C2); Rat heart tissue; Neonatal rat cardiomyocytes and mouse heart tissue; Human Heart Tissue; Aortic tissue | Up; Up; Up; Up; Up | Tong et al. 2015; Gryshkova et al. 2018; Wang et al. 2023; Wang et al. 2017; Raitoharju et al. 2011 | No |
| hsa-miR-29a-5p | Rat ventricular cells (H9C2) cells; Human Cardiac Tissue; Plasma | Up; Down; Up | Chen et al. 2019; Wang et al. 2017; Roncarati et al. 2014 | Yes |
| hsa-miR-34a-5p | Rat cardiac progenitor cells, Rat ventricular cells (H9C2), rat fibroblasts and rat aortic endothelial cells; Aortic tissue; Plasma | Up; Up; Up; Up | Piegari et al. 2016; Raitoharju et al. 2011; Ruggeri et al. 2018 | No |
| hsa-miR-96-5p | Murine heart tissue; Cardiomyocytes | Up; Up | Wang et al. 2021; Gryshkova et al. 2022 | Yes |
| hsa-miR-126-3p | Cardiac microvascular Endothelial cells; Cardiomyocytes; Plasma | Up; Up; Up; Up | Yang et al. 2017; Gryshkova et al. 2022; Frères et al. 2018; Martinez-Arroyo et al. 2023 | Yes |
| hsa-miR-133b | Neonatal rat cardiomyocytes; Plasma | Up; Up | Pan et al. 2018; Rigaud et al 2016 |  |
| hsa-miR-146a-5p | Cardiomyocytes; neonatal rat cardiomyocytes; Aortic Tissue; Plasma | Down; Up; Up; Up | Holmgren et al. 2016; Horie et al. 2010; Raitoharju et al. 2011; Rigaud et al 2016 | No |
| hsa-miR-146b-5p | Rat ventricular cells (H9C2); Mouse Aortic Smooth Muscle Cells; Cardiomyocytes | Up; Up; Up | Di et al. 2017; Sun et al. 2020; Gryshkova et al. 2022 | Yes |
| hsa-miR-182-5p | Cardiomyocytes | Up; Up | Chaudhari et al. 2016; Gryshkova et al. 2022 | Yes |
| hsa-miR-184 | Cardiomyocytes | Up | Gryshkova et al. 2022 | Yes |
| hsa-miR-185-5p | Cardiomyocytes; Isolated human cardiomyocytes; | Up; Down | Gryshkova et al. 2022; Sun et al. 2022 | Yes |
| hsa-miR-187-3p | Cardiomyocytes | Up; Up | Chaudhari et al. 2016; Gryshkova et al. 2022 | Yes |
| hsa-miR-208b-3p | Heart tissue; Plasma | Up; Up | Bronze-Da-Rocha et al. 2014; Boštjančič et al. 2010; Li et al. 2013; Widera et al. 2011 | Yes |
| hsa-miR-320a-3p | Mice heart tissue; Plasma | Up; Up | Yin et al. 2016; Oatmen et al. 2018 | No |
| hsa-miR-365a-5p | Cardiomyocytes; Neonatal mice ventricular cardiomyocytes; Plasma | Up; Up; Up | Gryshkova et al. 2022; Wu et al. 2017; Wu et al. 2021 | Yes |

**Supplementary Table 3:** **miRNAs upregulation per concentration tested following 72-hour exposure to structural cardiotoxicants**.

| **Treatments**  **(+ conc - µM)**  **miRNAs** | | | hsa-miR-126-3p | hsa-miR-146b-5p | hsa-miR-185-3p | hsa-miR-182-5p | hsa-miR-187-3p | hsa-miR-29a-5p | hsa-miR-365a-5p | hsa-miR-96-5p | hsa-miR-133b | hsa-miR-7-5p | hsa-miR-184 | hsa-miR-208b-3p |
| --- | --- | --- | --- | --- | --- | --- | --- | --- | --- | --- | --- | --- | --- | --- |
| **Anthracyclines and anthracycline-like drugs** | **Doxorubicin** | **3** | 0.7 | 0.3 | 3.2 | 2.1 | 2.5 | 6.1 | 2.7 | 0.7 | 5.3 | 3.4 | ND | 1.9 |
|  |  | **1** | 0.9 | 0.8 | 2.8 | 1.5 | 1.8 | 2.3 | 1.1 | 1.6 | 5.6 | 2.8 | ND | 1.8 |
|  |  | **0.3** | 0.1 | 0.6 | 0.9 | 29.4 | 3.4 | 0.2 | 0.0 | 40.0 | 0.5 | 6.6 | ND | 1.3 |
|  |  | **0.1** | 2.0 | 2.5 | 3.4 | 12.4 | 27.2 | 0.7 | 3.6 | 8.1 | 0.3 | 4.0 | 0.2 | 0.8 |
|  |  | **0.03** | 1.3 | 5.8 | 0.2 | 0.8 | 32.7 | 1.1 | 1.8 | ND | 1.2 | 1.1 | ND | 0.8 |
|  |  | **0.01** | 9.5 | ND | ND | 29.2 | 8.4 | ND | 3.9 | ND | 2.2 | 0.1 | ND | 0.04 |
|  |  | **0.003** | 4.4 | 0.8 | 5.7 | 5.9 | 7.3 | ND | 2.0 | ND | 2.7 | 0.04 | ND | 0.5 |
|  | **Daunorubicin** | **3** | 1.6 | 8.5 | 0.1 | ND | 3.0 | ND | ND | ND | 1.9 | 0.7 | 3.7 | 1.8 |
|  |  | **1** | 0.7 | 2.5 | 0.8 | ND | 1.4 | ND | 4.3 | 6.2 | 0.4 | 1.0 | 0.8 | 1.0 |
|  |  | **0.3** | 1.4 | 2.8 | 0.4 | 0.3 | 3.3 | 5.1 | 6.6 | 12.4 | 2.1 | 2.5 | 0.6 | 0.6 |
|  |  | **0.1** | 1.0 | 1.7 | 2.1 | 0.03 | 15.2 | 1.0 | 2.7 | 23.3 | 0.5 | 1.6 | 0.6 | 0.5 |
|  | **Idarubicin** | **3** | 1.1 | 3.4 | ND | 0.7 | 1.9 | 3.0 | 3.4 | 0.8 | 10.9 | 0.2 | 3.4 | 2.0 |
|  |  | **1** | 0.7 | 2.0 | 1.0 | 0.4 | 2.9 | 1.2 | 1.1 | 53.4 | 1.2 | 3.5 | 0.7 | 0.8 |
|  |  | **0.3** | 1.3 | 7.3 | 1.3 | 0.1 | 8.5 | 4.4 | 2.1 | 21.3 | 2.5 | 5.8 | 1.1 | 0.6 |
|  |  | **0.1** | 1.0 | 3.5 | 1.6 | 0.03 | 9.0 | 1.2 | 3.5 | 9.0 | 0.5 | 4.5 | 1.0 | 0.5 |
|  | **Epirubicin** | **10** | ND | ND | ND | ND | ND | 4.1 | 55.3 | ND | 3.1 | 16.9 | 28.4 | 11.8 |
|  |  | **3** | 1.3 | ND | 8.6 | 5.0 | 8.3 | 1.3 | 7.8 | ND | 0.9 | 2.7 | 6.7 | 5.1 |
|  |  | **1** | 6.7 | 3.9 | 36.2 | 7.3 | 1.6 | 1.1 | 6.9 | ND | 1.7 | 3.5 | 6.8 | 4.9 |
|  |  | **0.3** | 3.0 | 3.8 | 24.6 | 74.4 | 5.1 | 1.6 | 12.5 | ND | 1.8 | 12.1 | 5.2 | 4.9 |
|  | **Mitoxantrone** | **3** | 0.6 | 4.8 | 46.7 | ND | 16.7 | ND | ND | 3.7 | 41.9 | 0.3 | 7.6 | 0.1 |
|  |  | **1** | 0.7 | 5.4 | 1.5 | 2.4 | 1.9 | ND | 5.3 | 2.5 | 7.7 | 0.8 | 2.7 | 1.6 |
|  |  | **0.3** | 1.0 | 4.7 | 0.1 | 8.6 | 7.3 | ND | 1.4 | 4.4 | 3.1 | 1.0 | 1.0 | 0.4 |
|  |  | **0.1** | 1.0 | 2.9 | 1.6 | 3.0 | 6.8 | 1.8 | 0.9 | 5.2 | 1.4 | 1.6 | 1.2 | 0.7 |
| **Microtubular Dysruptors** | **Vincristine** | **0.3** | 1.4 | 0.3 | 1.1 | 0.4 | 0.7 | 1.0 | ND | 0.8 | 0.3 | 1.0 | ND | 2.2 |
|  |  | **0.03** | 1.3 | 0.4 | 1.0 | ND | 1.4 | 1.0 | 1.0 | 0.3 | 0.4 | 0.9 | ND | 1.6 |
|  |  | **0.003** | 1.9 | 0.7 | 2.0 | 0.5 | 1.9 | 0.7 | 1.6 | 0.6 | 0.2 | 1.1 | ND | 1.3 |
|  |  | **0.0003** | 3.2 | 1.2 | 1.4 | 0.4 | 1.3 | 0.4 | 0.6 | 0.3 | 0.3 | 1.0 | ND | 0.8 |
|  | **Vinblastine** | **0.3** | 4.3 | 11.8 | 2.7 | 2.1 | 3.1 | 0.2 | 3.3 | ND | 2.1 | 0.7 | ND | 1.1 |
|  |  | **0.03** | 5.2 | 7.6 | 0.1 | 1.1 | 37.8 | ND | 1.5 | 20.4 | 2.4 | 1.0 | ND | 1.2 |
|  |  | **0.003** | 5.8 | ND | 7.3 | ND | 11.5 | 0.6 | 0.9 | 5.5 | 12.7 | 0.3 | ND | 0.6 |
|  |  | **0.0003** | 4.9 | 1.4 | 3.9 | ND | ND | ND | 0.9 | ND | 7.1 | 0.6 | ND | 0.6 |
|  | **Vinorelbine** | **3** | 0.2 | 0.2 | 0.6 | ND | 0.3 | 0.9 | ND | ND | 0.1 | 1.8 | ND | 0.8 |
|  |  | **1** | 0.6 | 0.1 | 0.9 | 0.1 | 0.8 | 0.2 | 0.1 | 0.9 | 0.3 | 0.9 | ND | 0.7 |
|  |  | **0.3** | 2.0 | 1.1 | 1.9 | ND | 0.9 | 1.1 | 2.4 | 4.8 | 0.2 | 1.4 | ND | 1.7 |
|  |  | **0.1** | 1.4 | 3.1 | 1.2 | 0.7 | 1.1 | 0.4 | ND | 1.3 | 0.1 | 1.0 | ND | 0.8 |
|  | **Paclitaxel** | **10** | 1.2 | 0.6 | 1.9 | 0.3 | 0.4 | 4.2 | ND | 1.8 | 1.2 | 0.8 | 0.7 | 0.9 |
|  |  | **3** | 1.5 | 1.6 | 1.1 | 0.01 | 0.6 | 0.2 | ND | 1.4 | 3.7 | 0.7 | 1.0 | 1.0 |
|  |  | **1** | 2.0 | 0.4 | 0.8 | 0.01 | 0.7 | 1.3 | ND | 4.8 | 1.2 | 0.9 | 0.7 | 0.9 |
|  |  | **0.3** | 1.7 | 0.3 | 1.0 | 0.01 | 1.3 | 0.6 | ND | 6.0 | 9.0 | 0.8 | 0.7 | 1.0 |
|  | **Endothelin-1** | **0.1** | 3.2 | 1.8 | 0.02 | 0.7 | 3.8 | 0.4 | 0.6 | ND | 2.7 | 0.8 | ND | 1.6 |
|  |  | **0.03** | 2.7 | 2.8 | ND | 2.1 | 5.3 | ND | ND | ND | 3.5 | 0.8 | ND | 1.5 |
|  |  | **0.001** | 13.6 | 8.0 | 0.4 | 2.9 | 9.7 | ND | ND | ND | 2.7 | 0.4 | ND | 1.1 |
|  |  | **0.0003** | 2.0 | 16.5 | 1.3 | ND | 34.9 | ND | 1.2 | ND | 3.1 | 1.0 | ND | 1.0 |

| **Treatments**  **(+ conc - µM)**  **miRNAs** | | | hsa-miR-126-3p | hsa-miR-146b-5p | hsa-miR-185-3p | hsa-miR-182-5p | hsa-miR-187-3p | hsa-miR-29a-5p | hsa-miR-365a-5p | hsa-miR-96-5p | hsa-miR-133b | hsa-miR-7-5p | hsa-miR-184 | hsa-miR-208b-3p |
| --- | --- | --- | --- | --- | --- | --- | --- | --- | --- | --- | --- | --- | --- | --- |
| **Tyrosine Kinase Inhibitors** | **Sunitinib** | **1** | 1.9 | 0.7 | 0.2 | 0.7 | 0.4 | 0.7 | 0.8 | ND | 0.2 | 1.2 | ND | 1.7 |
|  |  | **0.3** | 0.6 | 0.6 | 0.9 | 1.0 | 0.3 | 0.8 | 0.7 | ND | ND | 1.1 | ND | 1.9 |
|  |  | **0.1** | 3.4 | 3.7 | 0.8 | 0.9 | 1.1 | 1.1 | 3.7 | ND | 0.2 | 1.5 | ND | 1.2 |
|  |  | **0.01** | 1.1 | 3.1 | 0.8 | 0.8 | 1.1 | 0.9 | 0.9 | ND | ND | 1.6 | ND | 1.7 |
|  | **Erlotinib** | **7.7** | 0.9 | 0.2 | 4.6 | 2.6 | 1.1 | 1.0 | 2.1 | 0.5 | 0.7 | 3.6 | ND | 1.0 |
|  |  | **2.3** | 1.0 | 1.1 | 5.4 | 1.2 | 0.9 | 2.0 | 13.3 | 0.6 | 0.1 | 4.1 | ND | 1.4 |
|  |  | **0.77** | 2.0 | 4.5 | 1.1 | 0.9 | 1.1 | 1.1 | 0.1 | 1.1 | 0.9 | 2.3 | ND | 4.5 |
|  |  | **0.23** | 2.0 | 0.4 | 0.5 | 0.1 | 0.5 | 0.3 | 0.4 | 0.2 | ND | 1.6 | ND | 3.6 |
|  | **Nilotinib** | **1** | 5.5 | 0.3 | 0.3 | 0.4 | 0.7 | 0.4 | ND | 1.4 | 0.3 | 0.9 | ND | 1.2 |
|  |  | **0.3** | 4.3 | 0.2 | 0.7 | 0.7 | 0.5 | 0.2 | 0.7 | ND | 0.4 | 0.5 | ND | 0.5 |
|  |  | **0.1** | 3.0 | 0.2 | 1.5 | 0.2 | 0.7 | 0.2 | 0.2 | 0.3 | 0.2 | 1.2 | ND | 1.1 |
|  |  | **0.01** | 1.4 | 0.2 | 0.7 | 0.3 | 0.7 | 0.5 | 0.8 | ND | 0.3 | 1.2 | ND | 1.0 |
|  | **Imatinib** | **10** | 1.0 | 0.8 | 4.7 | 0.3 | 0.8 | 0.5 | 9.2 | 2.8 | 0.8 | 1.0 | 1.2 | 1.1 |
|  |  | **3** | 2.1 | 0.8 | 2.2 | 0.01 | 0.7 | 1.0 | 2.4 | 2.8 | 1.8 | 0.6 | 1.0 | 0.9 |
|  |  | **1** | 2.6 | 0.6 | 1.5 | 0.01 | 0.7 | ND | 1.4 | 4.3 | 1.0 | 0.8 | 0.9 | 1.0 |
|  |  | **0.3** | 2.0 | 0.6 | 4.4 | 0.01 | 0.7 | 0.2 | ND | 12.2 | 2.4 | 0.7 | 1.0 | 1.0 |
|  | **Lapatinib** | **3** | 3.4 | 4.0 | 4.3 | 0.4 | 3.9 | ND | ND | ND | ND | 0.7 | 0.8 | 1.6 |
|  |  | **1** | 4.2 | 1.2 | 0.9 | 0.9 | 2.0 | ND | 1.9 | ND | ND | 0.3 | 0.6 | 1.2 |
|  |  | **0.3** | 50.7 | 12.9 | 0.6 | 1.0 | 2.7 | ND | ND | ND | ND | 0.5 | 1.1 | 1.6 |
|  |  | **0.1** | 2.1 | 3.2 | 0.9 | 3.2 | 4.5 | 0.1 | 0.5 | ND | ND | 0.3 | 0.6 | 1.0 |
|  | **Dasatinib** | **10** | 1.8 | 0.4 | 3.3 | 0.4 | 0.6 | 7.4 | 3.9 | 0.5 | 1.1 | 0.5 | 0.7 | 1.6 |
|  |  | **3** | 1.1 | 6.4 | 7.9 | 0.8 | 1.4 | 4.0 | 0.7 | ND | 3.3 | 0.9 | 0.8 | 1.2 |
|  |  | **1** | 2.1 | 0.9 | 1.5 | 0.9 | 0.5 | 3.6 | 0.8 | 4.7 | 1.6 | 0.8 | 0.8 | 1.2 |
|  |  | **0.3** | 1.6 | 0.8 | 6.4 | 2.5 | 1.7 | ND | 5.4 | 9.0 | 2.7 | 1.4 | 0.8 | 1.2 |
| **Proteasome Inhibitors** | **Bortezomib** | **0.3** | 8.5 | 1.3 | 3.3 | 22.6 | 2.4 | ND | ND | ND | ND | 5.3 | 1.5 | 0.7 |
|  |  | **0.1** | 7.7 | 0.5 | 1.8 | 27.5 | 1.9 | ND | ND | ND | ND | 5.1 | 1.2 | 1.0 |
|  |  | **0.03** | 13.2 | 1.7 | 2.1 | 16.1 | 2.6 | 0.7 | 0.5 | ND | ND | 1.8 | 1.0 | 0.7 |
|  |  | **0.01** | 17.8 | 1.0 | 2.3 | 3.4 | 2.4 | 0.8 | ND | ND | ND | 2.1 | 0.8 | 1.3 |
|  | **Ixazomib** | **1** | 3.1 | 3.2 | 0.9 | 5.6 | 2.4 | 4.7 | ND | 13.4 | 0.6 | 12.6 | 3.2 | 0.9 |
|  |  | **0.3** | 2.7 | 7.1 | 2.0 | 8.5 | 1.6 | 1.7 | 2.5 | 13.0 | 0.4 | 6.9 | 1.5 | 0.8 |
|  |  | **0.1** | 1.8 | 1.9 | 0.4 | 34.9 | 3.1 | 2.0 | 44.3 | 6.4 | 25.3 | 0.3 | 5.2 | 0.0 |
|  |  | **0.03** | 1.7 | 1.0 | 1.0 | 1.4 | 1.1 | 1.5 | ND | 8.8 | 1.2 | 2.0 | 0.8 | 0.9 |
|  | **Carfilzomib** | **10** | 0.4 | 0.01 | 0.2 | 5.7 | 20.5 | 0.8 | 0.02 | 1.6 | 0.3 | 3.8 | 1.6 | 1.0 |
|  |  | **3** | 0.5 | 0.06 | 3.3 | 14.0 | 2.1 | ND | 0.04 | 10.7 | 0.4 | 2.3 | 1.7 | 1.0 |
|  |  | **1** | 0.9 | 0.08 | 9.0 | 20.8 | 1.7 | 1.4 | 0.01 | 5.6 | 0.3 | 1.3 | 1.9 | 1.0 |
|  |  | **0.3** | 2.1 | 0.3 | 1.1 | 12.6 | 0.7 | 0.4 | 0.2 | 21.0 | 5.5 | 8.1 | 2.2 | 1.1 |

| **Treatments**  **(+ conc - µM)**  **miRNAs** | | | hsa-miR-126-3p | hsa-miR-146b-5p | hsa-miR-185-3p | hsa-miR-182-5p | hsa-miR-187-3p | hsa-miR-29a-5p | hsa-miR-365a-5p | hsa-miR-96-5p | hsa-miR-133b | hsa-miR-7-5p | hsa-miR-184 | hsa-miR-208b-3p |
| --- | --- | --- | --- | --- | --- | --- | --- | --- | --- | --- | --- | --- | --- | --- |
| **Drugs with Other MoA** | **BMS-986094** | **3** | 5.2 | 1.4 | 8.2 | 5.5 | ND | 0.5 | 0.7 | ND | 1.3 | 1.3 | ND | 1.0 |
|  |  | **1** | 2.8 | 11.3 | 3.3 | 7.1 | 8.8 | 1.2 | 1.3 | ND | 2.0 | 1.3 | ND | 1.1 |
|  |  | **0.3** | 2.9 | 4.2 | 0.5 | 8.8 | 2.7 | 1.0 | 0.7 | ND | 3.3 | 1.0 | ND | 1.3 |
|  |  | **0.1** | 0.3 | 1.8 | ND | 1.0 | 0.5 | 6.3 | 2.2 | ND | 1.3 | 1.7 | ND | 1.7 |
|  | **Milrinone** | **10** | 1.7 | 3.0 | 0.9 | 1.3 | 3.3 | 0.3 | 1.1 | ND | 2.5 | 1.3 | ND | 2.4 |
|  |  | **3** | 0.4 | 5.0 | 16.3 | 0.9 | 1.2 | ND | 0.4 | ND | 3.5 | 0.5 | ND | 2.1 |
|  |  | **0.3** | 5.9 | 19.0 | 0.2 | 1.6 | 3.7 | 0.02 | 4.9 | ND | 2.6 | 1.1 | ND | 1.8 |
|  |  | **0.1** | 7.7 | 3.6 | ND | 1.9 | ND | 0.6 | 3.7 | ND | 2.2 | 1.1 | ND | 1.3 |
|  | **Arsenic Trioxide** | **0.48** | 2.5 | 5.5 | ND | 2.0 | 5.0 | 0.3 | 1.6 | ND | 2.3 | 0.6 | ND | 2.0 |
|  |  | **0.16** | 1.8 | 2.7 | ND | 1.5 | 3.2 | 0.1 | 0.9 | ND | 2.8 | 0.8 | ND | 2.0 |
|  |  | **0.048** | 5.4 | 18.9 | 16.4 | 2.9 | 6.5 | 0.4 | 2.5 | ND | 1.1 | 1.0 | ND | 1.0 |
|  |  | **0.016** | 5.4 | 22.6 | 0.3 | 4.7 | 3.9 | 0.3 | 0.7 | ND | 2.4 | 1.5 | ND | 1.6 |
|  | **Tegaserod** | **0.3** | 3.2 | 0.9 | 1.0 | 15.7 | 3.7 | ND | ND | ND | ND | 2.0 | 0.7 | 1.4 |
|  |  | **0.1** | 0.9 | 1.2 | 0.9 | 1.9 | 2.0 | ND | ND | ND | ND | 1.3 | 0.9 | 1.7 |
|  |  | **0.03** | 4.9 | 0.7 | 1.3 | 3.9 | 2.0 | ND | ND | ND | ND | 1.4 | 0.8 | 1.2 |
|  |  | **0.01** | 2.0 | 0.5 | 0.7 | 1.8 | 2.5 | ND | ND | ND | ND | 0.8 | 0.8 | 1.8 |
|  | **Dexfenfluramine** | **0.3** | 0.5 | 0.7 | 0.7 | 0.01 | 0.6 | 0.7 | 8.9 | 0.7 | 0.1 | 1.0 | 0.8 | 0.9 |
|  |  | **0.1** | 0.6 | 0.4 | 1.0 | 0.003 | 0.7 | ND | ND | ND | 0.5 | 0.7 | 0.8 | 0.8 |
|  |  | **0.03** | 2.3 | 0.7 | 1.2 | 0.004 | 0.7 | 0.8 | 2.6 | 0.8 | 0.7 | 0.8 | 0.9 | 1.0 |
|  |  | **0.01** | 1.3 | 0.5 | 2.1 | 0.01 | 0.6 | 1.8 | ND | 1.8 | 4.0 | 0.8 | 1.2 | 1.1 |
|  | **Pergolide** | **10** | 2.0 | 1.1 | 1.9 | 2.2 | 0.6 | ND | ND | ND | ND | 1.0 | 1.4 | 2.5 |
|  |  | **3** | 1.1 | 20.8 | 7.2 | 0.9 | 21.8 | ND | ND | ND | ND | 1.4 | 1.7 | 2.6 |
|  |  | **1** | 1.1 | 6.1 | 5.6 | 2.2 | 0.9 | 0.2 | 5.2 | 5.6 | ND | 0.7 | 1.1 | 2.2 |
|  |  | **0.3** | 1.2 | 4.3 | 1.1 | 0.5 | 0.6 | 1.0 | ND | 2.8 | ND | 1.0 | 0.9 | 1.5 |
| **Drugs with effect on DNA/RNA integrity** | **5-FluoroUracil** | **10** | ND | 1.6 | ND | ND | 0.5 | 0.8 | ND | ND | ND | 0.7 | NA | ND |
|  |  | **3** | ND | 0.3 | ND | ND | 1.1 | 0.5 | ND | ND | ND | 0.5 | NA | ND |
|  |  | **1** | ND | 0.3 | ND | ND | 1.2 | 0.5 | ND | ND | ND | 0.5 | NA | ND |
|  |  | **0.3** | ND | 0.6 | ND | ND | 1.2 | 0.6 | ND | ND | ND | 0.7 | NA | ND |
|  | **Etoposide** | **10** | 0.9 | 3.0 | 0.3 | 1.1 | 4.5 | ND | ND | 0.9 | ND | 0.9 | 0.7 | 0.6 |
|  |  | **3** | 1.1 | 10.4 | 0.6 | 0.1 | 0.6 | 3.0 | 15.7 | 1.4 | 1.2 | 2.2 | 0.9 | 0.8 |
|  |  | **1** | 0.6 | 1.2 | 0.7 | 0.2 | 1.3 | ND | ND | 0.4 | 8.6 | 5.7 | 1.4 | 1.3 |
|  |  | **0.3** | 0.6 | 1.9 | 1.3 | 0.1 | 1.0 | 0.1 | 0.5 | 0.8 | 1.6 | 0.9 | 1.0 | 1.0 |
|  | **Pentamidine** | **3** | 1.1 | 1.5 | 1.0 | 0.7 | 1.1 | 0.8 | 1.0 | ND | 0.1 | 1.8 | ND | 1.8 |
|  |  | **1** | 0.8 | 1.0 | 1.9 | 0.4 | 1.0 | 0.9 | 0.4 | ND | ND | 1.3 | ND | 2.1 |
|  |  | **0.3** | 2.9 | 4.3 | 2.2 | 0.9 | 1.4 | 1.3 | 1.5 | ND | 0.2 | 2.0 | ND | 1.8 |
|  |  | **0.1** | 3.0 | 2.8 | 1.3 | 0.8 | 1.4 | 1.1 | 0.9 | ND | ND | 1.5 | ND | 1.9 |
|  | **Cyclophosphamide** | **100** | 1.2 | 7.1 | 1.3 | 2.4 | 0.8 | 1.5 | ND | ND | ND | 0.7 | 1.2 | 2.9 |
|  |  | **30** | 0.8 | 1.8 | 2.2 | 8.6 | 0.6 | 2.0 | ND | 9.6 | ND | 0.7 | 1.9 | 1.5 |
|  |  | **10** | 1.0 | 1.5 | 0.7 | 3.2 | 1.1 | 0.4 | ND | 6.8 | ND | 0.9 | 1.3 | 2.3 |
|  |  | **3** | 0.8 | 1.9 | 2.5 | 2.8 | 1.9 | ND | ND | 10.1 | ND | 1.0 | 1.3 | 2.0 |

Fold Changes (double normalization to housekeeping miRNAs and DMSO/vehicle control) ≥ 2 are considered biologically significant.

Reference:

1. Boštjančič, E. *et al.* (2010) ‘MicroRNA miR-1 is up-regulated in remote myocardium in patients with myocardial infarction’, *Folia Biologica*, 56(1), pp. 27–31.
2. Bronze-Da-Rocha, E. (2014) ‘MicroRNAs expression profiles in cardiovascular diseases’, *BioMed Research International*, 2014. doi: 10.1155/2014/985408.
3. Chaudhari, U. *et al.* (2016) ‘MicroRNAs as early toxicity signatures of doxorubicin in human-induced pluripotent stem cell-derived cardiomyocytes’, *Archives of Toxicology*, 90(12), pp. 3087–3098. doi: 10.1007/s00204-016-1668-0.
4. Chen, C. and Lu, Q. (2019) ‘miR ‐ 29a promotes pathological cardiac hypertrophy by targeting the PTEN / AKT / mTOR signalling pathway and suppressing autophagy’, (May), pp. 1–12. doi: 10.1111/apha.13323.
5. Di, Y. F. *et al.* (2017) ‘MiR-146b protects cardiomyocytes injury in myocardial ischemia/reperfusion by targeting Smad4’, *American Journal of Translational Research*, 9(2), pp. 656–663.
6. Frères, P. *et al.* (2018) ‘Variations of circulating cardiac biomarkers during and after anthracycline-containing chemotherapy in breast cancer patients’, *BMC Cancer*, 18(1), p. 102. doi: 10.1186/s12885-018-4015-4.
7. Gryshkova, V. *et al.* (2018) ‘miR-21-5p as a potential biomarker of inflammatory infiltration in the heart upon acute drug-induced cardiac injury in rats’, *Toxicology Letters*, 286(October 2017), pp. 31–38. doi: 10.1016/j.toxlet.2018.01.013.
8. Gryshkova, V. *et al.* (2022) ‘microRNAs signatures as potential biomarkers of structural cardiotoxicity in human ‑ induced pluripotent stem ‑ cell derived cardiomyocytes’, *Archives of Toxicology*, (0123456789). doi: 10.1007/s00204-022-03280-8.
9. Holmgren, G. *et al.* (2016) ‘Toxicology in Vitro MicroRNAs as potential biomarkers for doxorubicin-induced cardiotoxicity’, *TIV*, 34, pp. 26–34. doi: 10.1016/j.tiv.2016.03.009.
10. Horie, T. *et al.* (2010) ‘Acute doxorubicin cardiotoxicity is associated with miR-146a-induced inhibition of the neuregulin-ErbB pathway’, pp. 656–664. doi: 10.1093/cvr/cvq148.
11. Kaneto, C. M. *et al.* (2017) ‘MicroRNA pro fi ling identi fi es miR-7-5p and miR-26b-5p as differentially expressed in hypertensive patients with left ventricular hypertrophy’, 50, pp. 1–9. doi: 10.1590/1414-431X20176211.
12. Li, B. *et al.* (2014) ‘MicroRNA-7a / b Protects against Cardiac Myocyte Injury in Ischemia / Reperfusion by Targeting Poly ( ADP-Ribose ) Polymerase’, 9(3), pp. 1–9. doi: 10.1371/journal.pone.0090096.
13. Martinez-Arroyo, O. *et al.* (2023) ‘High miR-126-3p levels associated with cardiovascular events in a general population’, *European Journal of Internal Medicine*, 113(December 2022), pp. 49–56. doi: 10.1016/j.ejim.2023.04.013.
14. Oatmen, K. E. *et al.* (2018) ‘Identification of a novel microRNA profile in pediatric patients with cancer treated with anthracycline chemotherapy’, *Am J Physiol Heart Circ Physiol*, (315), pp. H1443–H1452. doi: 10.1152/ajpheart.00252.2018.
15. Pan, Y. L. *et al.* (2018) ‘MiR-133b-5p contributes to hypoxic preconditioning-mediated cardioprotection by inhibiting the activation of caspase-8 and caspase-3 in cardiomyocytes’, *Molecular Medicine Reports*, 17(5), pp. 7097–7104. doi: 10.3892/mmr.2018.8784.
16. Pang, L. *et al.* (2019) ‘Workshop Report’, *Circulation Research*, 125(9), pp. 855–867. doi: 10.1161/CIRCRESAHA.119.315378.
17. Piegari, E. *et al.* (2016) ‘MicroRNA-34a regulates doxorubicin-induced cardiotoxicity in rat’, *Oncotarget*, 7(38), pp. 62312–62326. doi: 10.18632/oncotarget.11468.
18. Raitoharju, E. *et al.* (2011) ‘MiR-21, miR-210, miR-34a, and miR-146a/b are up-regulated in human atherosclerotic plaques in the Tampere Vascular Study’, *Atherosclerosis*, 219(1), pp. 211–217. doi: 10.1016/j.atherosclerosis.2011.07.020.
19. Rigaud, V. O. *et al.* (2017) ‘Circulating miR-1 as a potential biomarker of doxorubicin- induced cardiotoxicity in breast cancer patients Study population’, *Oncotarget*, 8(4), pp. 6994–7002. doi: <https://doi.org/10.18632/oncotarget.14355>.
20. Roncarati, R. *et al.* (2013) ‘Circulating miR-29a, Among Other Up-Regulated MicroRNAs, Is the Only Biomarker for Both Hypertrophy and Fibrosis in Patients With Hypertrophic Cardiomyopathy’, *Journal of the American College of Cardiology*. doi: 10.1016/j.jacc.2013.09.041.
21. Ruggeri, C. *et al.* (2018) ‘Role of microRNAs in doxorubicin-induced cardiotoxicity: an overview of preclinical models and cancer patients’, *Heart Failure Reviews*, 23(1), pp. 109–122. doi: 10.1007/s10741-017-9653-0.
22. Sun, D. *et al.* (2020) ‘MiRNA 146b-5p protects against atherosclerosis by inhibiting vascular smooth muscle cell proliferation and migration’, *Epigenomics*, 12(24), pp. 2189–2204. doi: 10.2217/epi-2020-0155.
23. Sun, J. *et al.* (2022) ‘LncRNA ROR modulates myocardial ischemia-reperfusion injury mediated by the miR-185-5p / CDK6 axis’, *Laboratory Investigation*, 102(5), pp. 505–514. doi: 10.1038/s41374-021-00722-2.
24. Tong, Z. *et al.* (2015) ‘MiR-21 Protected Cardiomyocytes against Doxorubicin-Induced Apoptosis by Targeting BTG2’, 2, pp. 14511–14525. doi: 10.3390/ijms160714511.
25. Wang, J. *et al.* (2021) ‘MiR-96 promotes myocardial infarction-induced apoptosis by targeting XIAP’, *Biomedicine and Pharmacotherapy*, 138, p. 111208. doi: 10.1016/j.biopha.2020.111208.
26. Wang, Q. *et al.* (2023) ‘Heliyon miR-21-5p prevents doxorubicin-induced cardiomyopathy by downregulating BTG2’, *Heliyon*, 9(5), p. e15451. doi: 10.1016/j.heliyon.2023.e15451.
27. Wang, Y. *et al.* (2017) ‘Expression of Bcl-2 and microRNAs in cardiac tissues of patients with dilated cardiomyopathy’, *Molecular Medicine Reports*, 15(1), pp. 359–365. doi: 10.3892/mmr.2016.5977.
28. Widera, C. *et al.* (2011) ‘Diagnostic and prognostic impact of six circulating microRNAs in acute coronary syndrome’, *Journal of Molecular and Cellular Cardiology*, 51(5), pp. 872–875. doi: 10.1016/j.yjmcc.2011.07.011.
29. Wu, H. *et al.* (2017) ‘MicroRNA-365 accelerates cardiac hypertrophy by inhibiting autophagy via the modulation of Skp2 expression’, *Biochemical and Biophysical Research Communications*, 484(2), pp. 304–310. doi: 10.1016/j.bbrc.2017.01.108.
30. Wu, H. B. *et al.* (2021) ‘The expression of miR-365 in serum of hypertension patients with left ventricular hypertrophy was up-regulated, which was positively correlated with left ventricular mass index’, *Pharmacogenomics and Personalized Medicine*, 14(July), pp. 905–913. doi: 10.2147/PGPM.S319945.
31. Wu, J. *et al.* (2018) ‘Chemico-Biological Interactions Cardioprotective e ff ect of paeonol against epirubicin-induced heart injury via regulating miR-1 and PI3K / AKT pathway’, *Chemico-Biological Interactions*, 286(November 2017), pp. 17–25. doi: 10.1016/j.cbi.2018.02.035.
32. Yang, H. H. *et al.* (2017) ‘Protective Effects of MicroRNA-126 on Human Cardiac Microvascular Endothelial Cells Against Hypoxia/Reoxygenation-Induced Injury and Inflammatory Response by Activating PI3K/Akt/eNOS Signaling Pathway’, *Cellular Physiology and Biochemistry*, 42(2), pp. 506–518. doi: 10.1159/000477597.
33. Yin, Z. *et al.* (2016) ‘miR-320a mediates doxorubicin-induced cardiotoxicity by targeting VEGF signal pathway’, *Aging*, 8(1), pp. 192–207. doi: 10.18632/aging.100876.
